# Timeliness in the German surveillance system for infectious diseases: Amendment of the infection protection act in 2013 decreased local reporting time to 1 day

**DOI:** 10.1371/journal.pone.0187037

**Published:** 2017-10-31

**Authors:** Jakob Schumacher, Michaela Diercke, Maëlle Salmon, Irina Czogiel, Dirk Schumacher, Hermann Claus, Andreas Gilsdorf

**Affiliations:** Department of Infectious Disease Epidemiology, Robert Koch Institute, Berlin-Mitte, Berlin, Germany; Australian National University, AUSTRALIA

## Abstract

Time needed to report surveillance data within the public health service delays public health actions. The amendment to the infection protection act (IfSG) from 29 March 2013 requires local and state public health agencies to report surveillance data within one working day instead of one week. We analysed factors associated with reporting time and evaluated the IfSG amendment. Local reporting time is the time between date of notification and date of export to the state public health agency and state reporting time is time between date of arrival at the state public health agency and the date of export. We selected cases reported between 28 March 2012 and 28 March 2014. We calculated the median local and state reporting time, stratified by potentially influential factors, computed a negative binominal regression model and assessed quality and workload parameters. Before the IfSG amendment the median local reporting time was 4 days and 1 day afterwards. The state reporting time was 0 days before and after. Influential factors are the individual local public health agency, the notified disease, the notification software and the day of the week. Data quality and workload parameters did not change. The IfSG amendment has decreased local reporting time, no relevant loss of data quality or identifiable workload-increase could be detected. State reporting time is negligible. We recommend efforts to harmonise practices of local public health agencies including the exclusive use of software with fully compatible interfaces.

## Background

The German surveillance system aims at early identification of infections of communicable diseases in order to prevent further spreading. Time lags in notifying and reporting data from local to the state and national level result in a delay of public health response, including outbreak detection. Timely data reporting is crucial for the management of widespread outbreaks, which affect more than one local public health agency. Public health actions such as formation of an outbreak team, press work, explorative case interviews and epidemiological studies need to happen as fast as possible after the beginning of an outbreak in order to prevent harm. For example during the 2012 Germany Norovirus outbreak [1] timely reporting of surveillance data was essential for the identification of the source.

### Description of the German surveillance system for infectious diseases

Suspicion, illness or death of 29 communicable diseases and the laboratory confirmation of 52 pathogens are mandatorily notifiable by physicians and laboratories to local public health agencies according to §6.1.1 and §7.1 infection protection act (IfSG) [2]. Laboratories and physicians usually notify via telefax. At the local public health agency the notified event is investigated and infection control measures are initiated. All available case information is entered into notification software. If the information is consistent with case definitions issued by the Robert Koch Institute, the local public health agency reports anonymized information about the case to the state public health agency via an electronic system; from there data is reported to the Robert Koch Institute. To manage infectious disease data, the local public health agencies use a notification software developed by Robert Koch Institute (SurvNet*@*RKI*)* or other commercially available notification software products (Software A—E) [3]. All state public health institutes and the Robert Koch Institute use SurvNet@RKI to manage infectious disease data. The notification software products use specifications for data reporting defined by the Robert Koch Institute. In 2011 the Robert Koch Institute updated the specifications from version 2 to version 3. Automatic outbreak detection systems run daily [4].

### IfSG amendment

The 2011 Germany verotoxigenic *E*. *coli* (VTEC) O104:H4 outbreak [5] highlighted the necessity of a short reporting time. A study found a median reporting time from the notification of the local public health agency to the Robert Koch Institute of 7 days (Interquartile range: 4–11) before the outbreak [6].

After this outbreak, the infection protection act was revised on 29/03/2013. This IfSG amendment stipulated that local and state public health agencies need to report cases within one working day, instead of within one week. To comply with the new regulations no new software or update was needed. No additional personnel was required, as the process of reporting data is accomplished within a few minutes. The process of investigating a notified event did not need to change. The IfSG amendment also changed other parts of the law. Since then, persons who notify infectious diseases need to ensure that their notifications arrive within 24 hours at the local public health agency.

### Objectives of the study

We analysed factors that might be associated with reporting time, including the IfSG amendment, to evaluate changes and direct future research and interventions to improve the timeliness of reporting.

## Methods

### Data selection

We extracted all cases from the German national database for notifiable diseases that were reported according to § 7.1. or § 6.1.1. IfSG between 28/03/2012 and 28/03/2014. We excluded cases of mumps, rubella, chickenpox and pertussis that have only been mandatory notifiable since the IfSG amendment and cases of rare diseases (less than 10 cases in the specified time period). We analysed data from all local public health agencies that reported at least one case.

### Delay calculation

**Fig 1:**
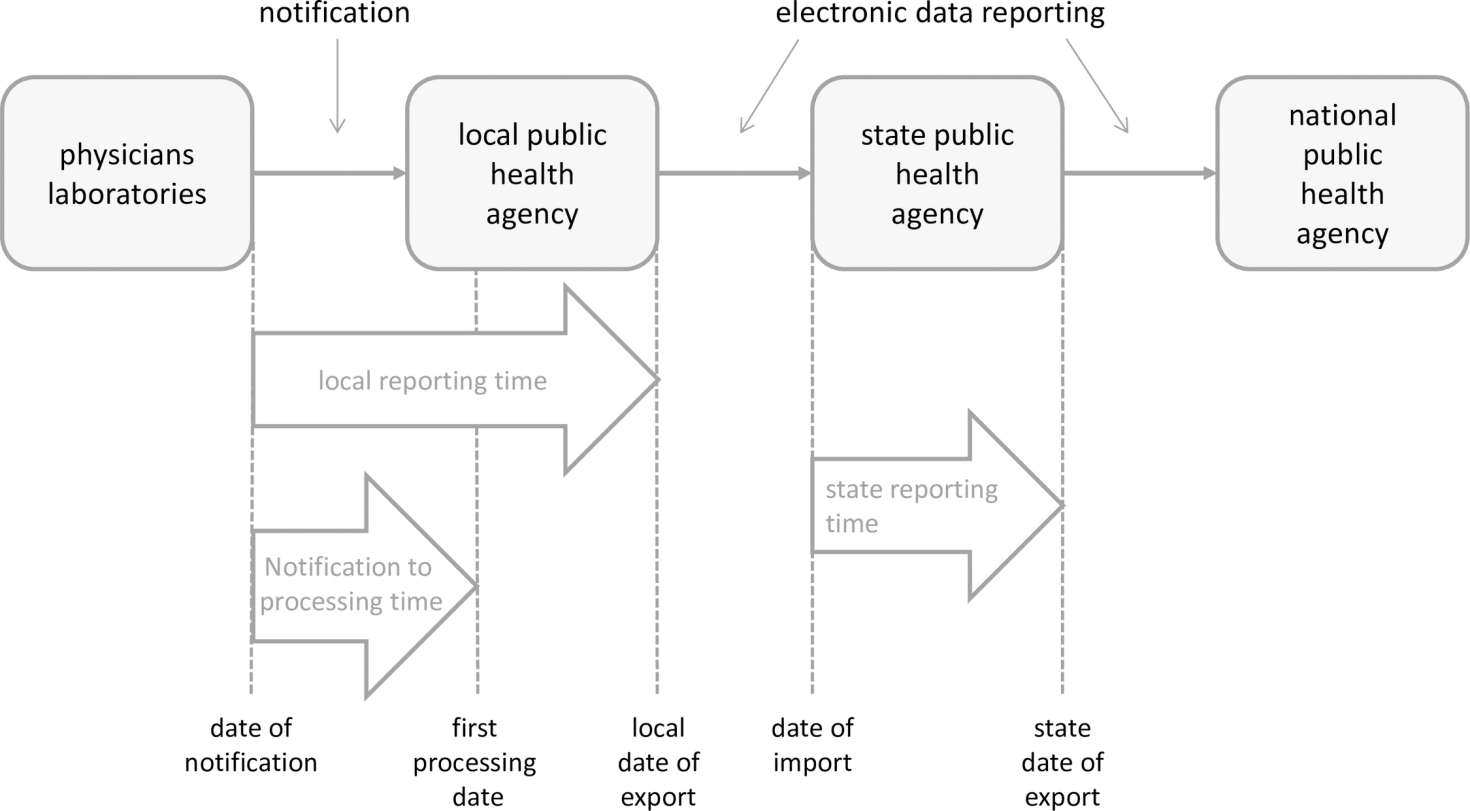
Sketch of German surveillance system. Dates (dotted lines) are available in the database at Robert Koch Institute.

Our analysis focuses on three delay periods that are defined as follows (Fig 1).

- We defined *local reporting time* as the time interval between date of notification and date of export to the state public health agency. The date of notification is the date when information about a case is sent to the local public health agency. If several dates exist for one event (e.g. multiple dates of notification from different sources), the earliest date was taken. The local public health agency manually enters this date into the software. The date of export is the date when the local public health agency reports the information to the state public health agency. The local reporting time includes several time periods:
  - the time period the notification takes from the notifier to the local public health agency
  - the time period from the arrival of the notification until the first processing date, which is the day when the investigation starts. This is usually the next working day after the information arrives at the local public health agency. For example: if a notification arrives on Saturday, the next working day would be a Monday.
  - the time period from the first processing date until the case definition is fulfilled
  - the time period from the date when the case definition is fulfilled until the date of export to the state public health agency
- We defined the *notification to processing time* as the time between the date of notification and the first processing date. This time period is a subset of the local reporting time. It is only computable for software products that work according to software specifications version 3. For this time period only notifications that were sent between a Monday and a Thursday were analysed, as these notifications are usually processed by the local public health agency on the next day.
- We defined the *state reporting time* as the time interval between date of arrival at the state public health agency and the date of export to the national public health agency.

For each delay period we excluded any negative values and values above 183 days (6 months), as they are presumably caused by incorrect data entry. Incorrect data entry can happen if a staff member enters wrong information into the software e.g. she or he sets the date of notification after the date of export. Implausible long time periods presumably come from typing errors when entering the year.

### Univariable analysis of potential determinants of reporting time

We calculated the median and the interquartile range (IQR) for the local and state reporting time in different subsets of the data.

To analyse local reporting time we computed the impact of the IfSG amendment (defined as first processing date before week 14 of 2013), disease category, software, age, sex, software specifications and the weekday of the date of notification. Furthermore we analysed different aspects of local public health agencies. We categorised them into four groups (fastest quartile to slowest quartile), according to their mean reporting time before the IfSG amendment. Also, we categorised local public health agencies according to the number of notifications per day.

We analysed state reporting time by disease category, weekday of notification arrival at the state public health agency and IfSG amendment.

For graphical analysis of the time periods we computed a bar chart with time in months on the x-axis and the mean reporting time with confidence intervals on the y-axis.

### Multivariable analysis of potential determinants of local reporting time

To adjust for confounding we fitted a negative binominal regression model with a log-link to the local reporting time (measured in days). As independent variables we chose the potential determinants disease category, software, period before and after IfSG amendment, weekday the date of notification and local public health agencies grouped into quartiles according to their mean number of notifications per day. Confidence intervals of the regression coefficients were calculated according to Wald [7], assuming asymptotic normality.

### Compliance with IfSG amendment

To assess compliance with the IfSG amendment we computed the percentage of cases that are reported from the local public health agency to the state public health agency or from the state public health agency to Robert Koch Institute within one working day. A working day was defined as a day from Monday to Friday that does not fall on a nationwide public holiday.

### IfSG amendment of notification process

To assess the change of the notification process by the IfSG amendment we analysed cases from local public health agencies that had reported at least 10 cases according to software specifications version 3 before the IfSG amendment. We calculated median and mean of the notification to processing time before and since the IfSG amendment. For graphical analysis we drew a bar chart with months from April 2012 until March 2014 on the x-axis and the mean time with confidence intervals on the y-axis.

### Data quality and workload before and since IfSG amendment

To evaluate a potential decrease in data quality we assessed the completeness of following variables: onset of disease and the employment status of a person: paid to work in the preparation of food or working in a communal facility.

Each individual case can be reported several times, as new information becomes available and case information is altered in the database. We use the mean number of reports of each case as proxy for the workload. Earlier reports could require more alterations of a case, which would be an additional workload for the staff of the local public health agencies.

The information on these quality and workload parameters is shown graphically over the months of the selected time period.

### Software and additional material

All statistical analysis was conducted using R [8]. Regression analysis was conducted using the MASS package [9]. The R-Script is available as Supporting Information (S1 File).

## Results

### Data selection

We analysed 665,442 cases that were processed by 392 local public health agencies and 16 state public health agencies. It was possible to calculate the local reporting time for 550,129 (83%) cases: dates of notification for 621,910 (93%) cases, and dates of export from the local public health agency for 590,371 (89%) cases were available respectively. It was possible to calculate the state reporting time for 606,063 (91%) cases: dates of arrival at the state public health agency were available for 606,089 (91%) cases, and dates of export from the state public health agency for 665.416 (100%) cases."

For the calculation of the local reporting time 401 cases were excluded because their reporting time was recorded as negative and 1,810 cases were excluded because their reporting time was above 183 days. For the calculation of the state reporting time 1,382 cases were excluded because their reporting time was recorded as negative and 19 cases were excluded because their reporting time was above 183 days.

### Analysis of local reporting time

**Fig 2:**
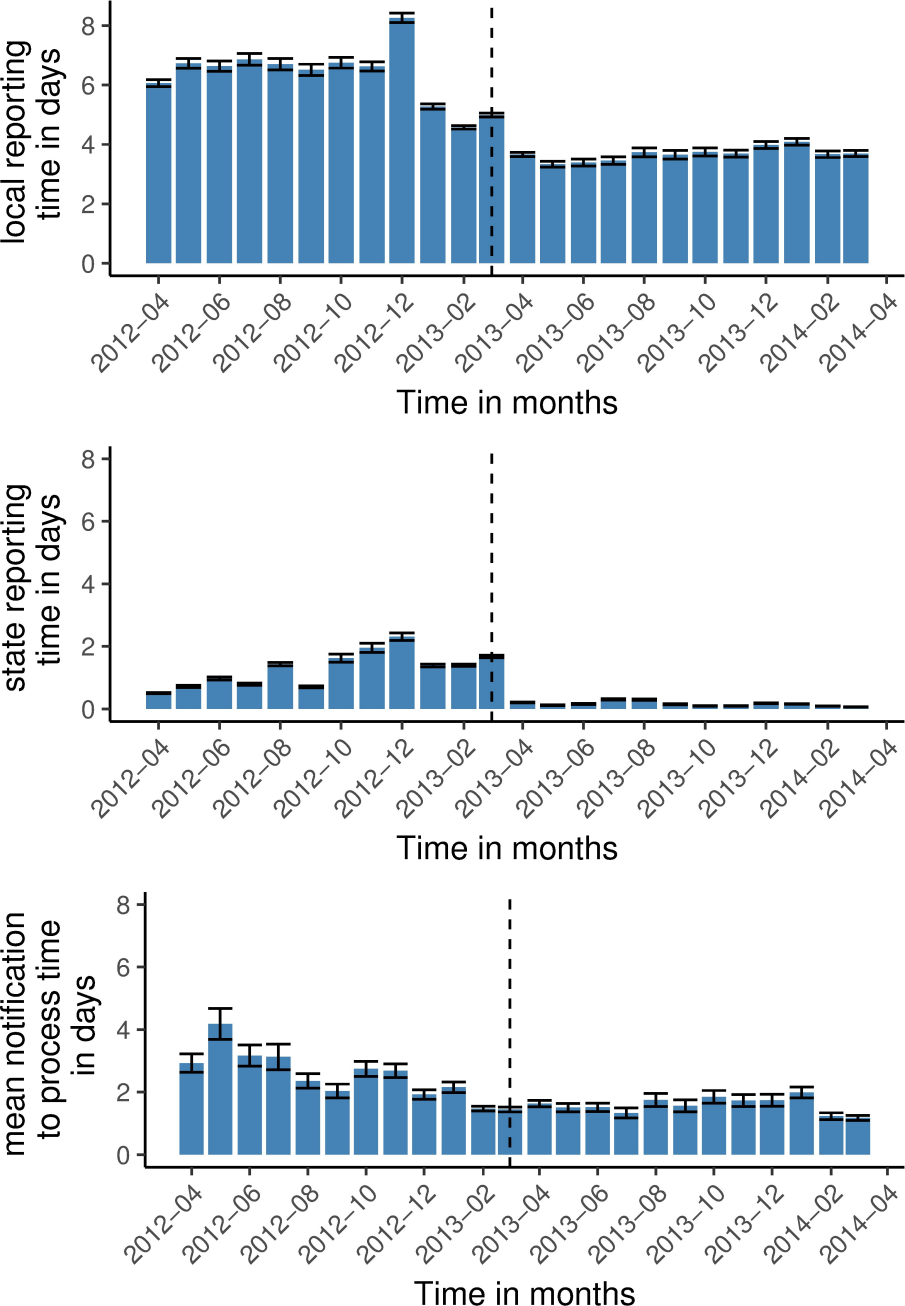
Mean time periods of reporting time per month. Local reporting time (interval between date of notification and date of export to the state public health agency), state reporting time (interval between date of arrival at the state public health agency and the date of export to the national public health agency), notification to process time (interval between the date of notification and the first processing date). Data from the German surveillance system for infectious diseases. Dotted line = date of amendment of infection protection act (29/03/2013). Error bars = 95% confidence intervals. Germany 2012–2014.

The median local reporting time was 4 days (IQR 2–7) before and 1 day (IQR 1–4) after the IfSG amendment (Fig 2 and Table 1). The multivariable model calculates a reporting time after the IfSG amendment that was 54% of the reporting time before the amendment (CI 54%– 55%).

**Table 1:** Univariable and multivariable analysis of factors associated with the reporting time from the local public health agencies to the state public health agency, Germany 2012–2014.

	Number of cases (%)		Median local reporting time in days(IQR)	Adjusted ratio of expected local reporting time (CI)
**IfSG amendment**				
before IfSG amendment	284,188		4 (2–7)	Ref.
since IfSG amendment	263,730		1 (1–4)	0.54 (0.54–0.55)
Local public health agencies, grouped into 4 by mean reporting time before IfSG amendment*				
fastest quartile	72,839 (28)		1 (1–3)	–
second fastest quartile	67,990 (26)		1 (1–3)	–
third fastest quartile	62,467 (25)		2 (1–4)	–
slowest quartile	55,896 (21)		3 (1–7)	–
Software used at local public health agency*				
SurvNet@RKI	49737 (19)		2 (1–5)	Ref.
Software A	19,628 (7)		1 (1–3)	1.00 (0.99–1.02)
Software B	32,241(12)		1 (1–4)	1.13 (1.12–1.15)
Software C	10,522 (4)		2 (1–5)	1.15 (1.13–1.17)
Software D	135,048(51)		1 (1–3)	1.05 (1.04–1.06)
Software E	15,472 (6)		3 (1–6)	1.81 (1.78–1.84)
Software Specifications*				
Software specifications version 2	178,515 (68)		1 (1–4)	Ref.
Software specifications version 3	84,133 (32)		2 (1–5)	1.37 (1.36–1.39)
Weekday of date of notification*				
Monday	67,469 (26)		1 (1–3)	Ref.
Tuesday	51,352 (19)		1 (1–3)	1.00 (0.99–1.01)
Wednesday	45,065 (17)		1 (1–2)	1.07 (1.06–1.08)
Thursday	47,878 (18)		1 (1–4)	1.10 (1.09–1.11)
Friday	43,067 (16)		3 (0–5)	1.30 (1.29–1.31)
Saturday	6,679 (3)		3 (2–4)	1.49 (1.46–1.52)
Sunday	2,220 (1)		2 (1–4)	1.39 (1.35–1.44)
Local public health agencies, grouped into 4, by numbers of notifications per working day*				
0–1.5 notifications	17,755 (7)	2 (1–4)		Ref.
1.5–2.5 notifications	43,732 (17)	2 (1–4)		1.02 (1.01–1.03)
2.5–4 notifications	68,795 (26)	1 (1–4)		0.94 (0.92–0.95)
4–23.5 notifications	133,448 (51)	1 (1–4)		0.94 (0.93–0.95)

Ref. = Reference group. CI = 95% confidence interval according to Wald, IQR = Interquartile range. Adjusted ratio of expected local reporting time = Exponentiated estimate of corresponding negative binomial model coefficient, fitted with local reporting time as dependent variable.

*Only data since the IfSG amendment is shown. Germany 29/03/2012-28/03/2014

#### Differences among local public health agencies

There were substantial differences among local public health agencies in the local reporting time. The median local reporting time of the slowest quartile of local public health agencies was 7 days before the IfSG amendment and 3 days after the IfSG amendment. The median local reporting time of the fastest quartile did change from a median of 2 days to a median of 1 day (Table 2.5 in S2 File).

#### Software

The reporting time was dependent on the software product used at the local public health agency. Cases reported with Software E had a 82% longer reporting time than cases reported with SurvNet@RKI. Cases reported according to software specifications version 3 had a 38% longer reporting time than those according to specifications version 2.

#### Weekday

Most notifications were done on Monday, the least on Saturday and Sunday. After the IfSG amendment the weekdays with the longest median reporting times were Friday (3 days; IQR 0–5 days, 30% longer reporting time than on Monday), Saturday (3 days; IQR 2–4 days, 49% longer reporting time than on Monday) and Sunday (2 days; IQR 1–4 days, 40% longer reporting time than on Monday). The IQR was bigger on Friday, showing that some cases were reported on Friday itself and some were reported on the following Monday.

#### Number of notifications per day

The local public health agencies that had more notifications per working day had a shorter reporting time (median 1 day) than those with fewer notifications per day (median 2 days).

#### Age and sex

There was no difference in the univariable analysis of reporting time needed for female or male cases and no relevant difference amongst different age groups (Table 2.9. in S2 File)

#### Diseases

The most frequent disease was Norovirus gastroenteritis with 230,664 cases, which is why it was used as a reference group for the multivariable model. The reporting time varies substantially between different diseases Table 2. A decrease in reporting time after the IfSG amendment was observable for all diseases (Table 2.10 in S2 File).

**Table 2:** Reporting time from the local public health agencies to the state public health agency by disease category, Germany 2012–2014.

Disease	Number of cases (%)	Median local reporting time in days(IQR)	Adjusted ratio of expected local reporting time (CI)
Brucellosis	73 (0.0%)	1.5 (1–4)	1.07 (0.81–1.41)
Campylobacteriosis	136,535 (20.5%)	1 (1–4)	0.94 (0.93–0.95)
Creutzfeld Jakob Disease (CJD)	262 (0.0%)	4 (2–12.75)	3.61 (3.15–4.14)
Cryptosporidiosis	3,243 (0.5%)	1 (1–4)	0.97 (0.93–1.01)
Dengue fever	1,581 (0.2%)	2 (1–4)	1.06 (1–1.13)
Diphtheria	14 (0.0%)	1 (1–1.5)	0.52 (0.24–1.1)
Epidemic keratoconjunctivitis	4,415 (0.7%)	2 (1–4)	1.11 (1.07–1.15)
Giardiasis	11,225 (1.7%)	1 (1–4)	1.08 (1.06–1.11)
*Haemophilus influenzae* invasive disease	858 (0.1%)	1 (1–4)	1.06 (0.98–1.15)
Hantavirus disease	2,769 (0.4%)	2 (1–6)	1.60 (1.51–1.7)
Hemolytic uremic syndrom (HUS)	149 (0.0%)	2 (1–4)	0.87 (0.71–1.06)
Hepatitis A	1,961 (0.3%)	2 (1–4)	1.03 (0.98–1.08)
Hepatitis B	4,685 (0.7%)	4 (1–10)	2.86 (2.77–2.96)
Hepatitis C	10,913 (1.6%)	4 (1–10)	2.93 (2.86–2.99)
Hepatitis D	124 (0.0%)	2 (1–6.5)	2.36 (1.93–2.87)
Hepatitis E	1,090 (0.2%)	2 (1–4)	1.41 (1.31–1.51)
Influenza	84,577 (12.7%)	1 (1–4)	0.72 (0.71–0.72)
Legionnaires' disease	1,770 (0.3%)	2 (1–5)	1.44 (1.36–1.52)
Leptospirosis	190 (0.0%)	1 (1–3)	0.86 (0.72–1.02)
Listeriosis	1,062 (0.2%)	2 (1–4)	1.33 (1.24–1.43)
Measles	2,097 (0.3%)	1 (1–3)	0.80 (0.76–0.84)
Meningococcal invasive disease	687 (0.1%)	2 (1–4)	1.30 (1.19–1.42)
Methicillin–resistant *Staphylococcus aureus*, *invasive infection*	8,771 (1.3%)	2 (1–5)	1.70 (1.66–1.74)
Norovirus gastroenteritis	230,664 (34.7%)	1 (1–4)	Ref.
Ornithosis	33 (0.0%)	2 (2–3)	0.85 (0.55–1.3)
Q fever	405 (0.1%)	2 (1–5)	1.40 (1.24–1.57)
Rotavirus gastroenteritis	92,046 (13.8%)	1 (1–4)	0.95 (0.94–0.96)
Salmonellosis	42,968 (6.5%)	1 (1–3)	0.94 (0.93–0.95)
Shigellosis	1,147 (0.2%)	1 (1–4)	0.92 (0.86–0.99)
Tick–borne encephalitis (TBE)	661 (0.1%)	2 (1–5)	1.56 (1.43–1.7)
Trichinosis	29 (0.0%)	4 (4–7)	2.15 (1.47–3.13)
Tuberculosis	8,593 (1.3%)	7 (2–18)	4.42 (4.32–4.53)
Tularemia	41 (0.0%)	1 (1–2.75)	0.95 (0.66–1.38)
Typhoid	155 (0.0%)	2 (1–3)	1.60 (1.34–1.91)
Chikungunya fever and VHF	28 (0.0%)	3 (1–4.25)	0.86 (0.56–1.31)
VTEC disease	4,066 (0.6%)	2 (1–4)	1.16 (1.12–1.2)
Yersiniosis	5,555 (0.8%)	1 (1–3.5)	0.95 (0.92–0.98)

Ref. = Reference group. CI = 95% confidence interval according to Wald, IQR = Interquartile range, VHF = Viral haemorrhagic fever, VTEC = verotoxigenic *E*. *coli*. Adjusted ratio of expected local reporting time = Exponentiated estimate of corresponding negative binomial model coefficient, fitted with local reporting time as dependent variable. Germany 29/03/2013-28/03/2014.

### Compliance with IfSG amendment

58% of cases were reported within one working day from the local public health agency to the state public health agency.

### Analysis of state reporting time

The median reporting time of the state public health agencies was 0 (IQR 0–1) before and 0 (IQR 0–0) after the IfSG amendment. A daily reporting of cases was already common practice before the IfSG amendment. The mean reporting time changed (Fig 2). There were only non-relevant differences amongst different federal states, amongst different weekdays and amongst different diseases (Table 2.12 in S2 File).

98% of cases were reported within one working day from the state public health agency to the national public health agency (Table 2.12 in S2 File).

### Analysis of notification to processing time

The median notification to process time before and after the IfSG amendment was 0 days. The mean changed from 2.26 days to 1.57 days (Table 2.13 S2 File). Graphically the reduction of the notification to process time took place in the year before the IfSG amendment (Fig 2).

### Quality and workload of data

**Fig 3:**
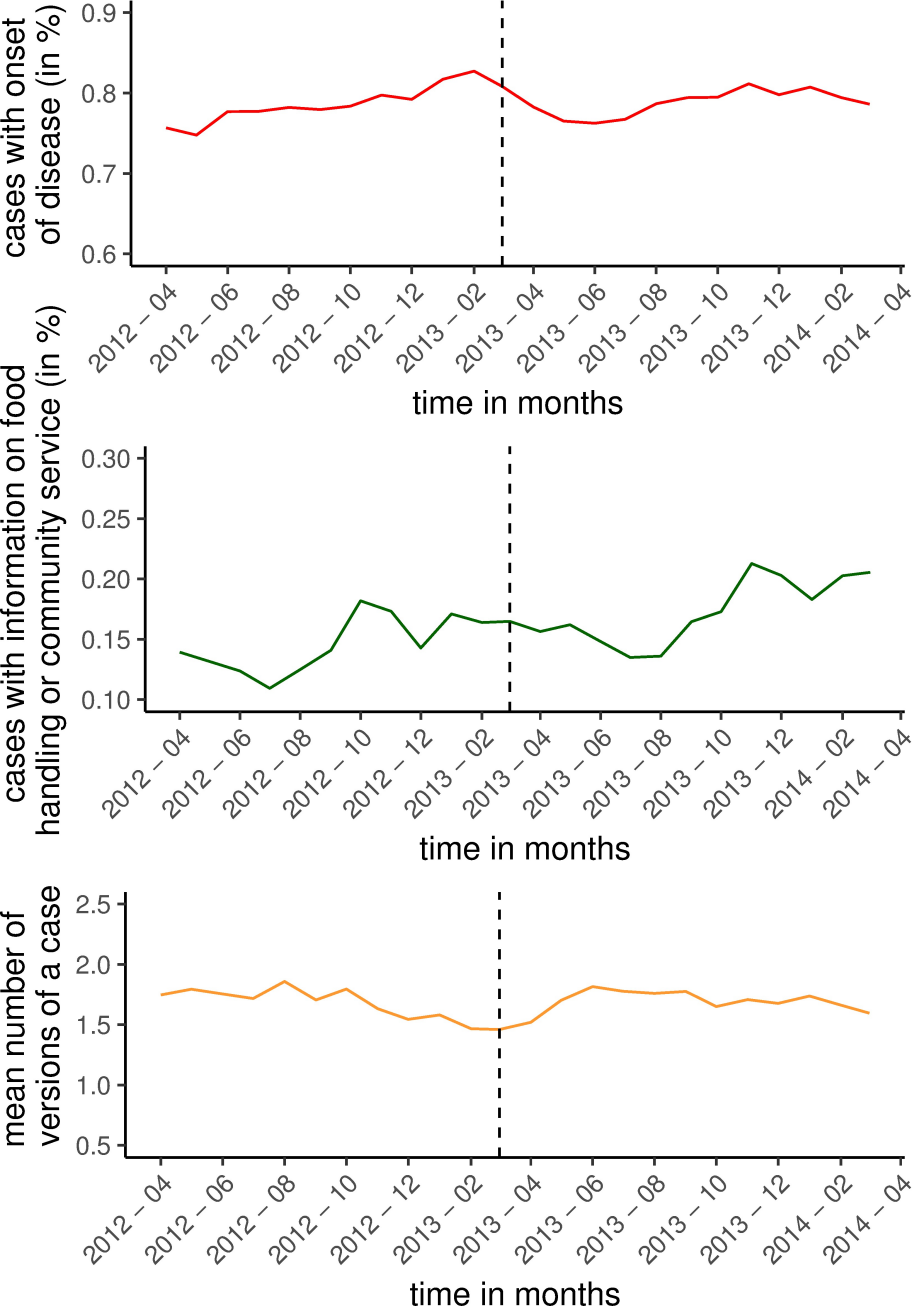
Graphical display of data quality and workload before and after IfSG amendment. Germany 2012–2014. Data from the German surveillance system for infectious diseases. Green: percentage of cases where information on the status community service is present, red: percentage of cases where information on onset of disease is available, yellow: mean number of versions. Dotted line = date of amendment of infection protection act (29/03/2013). Band = 95% confidence intervals.

Completeness of the employment status of a person: paid to work in the preparation of food or working in a communal facility is 15% before the IfSG amendment and 18% since the IfSG amendment. The percentage of cases where the onset of disease was available was 79% before and since the IfSG amendment. The mean numbers of versions per case was 1.62 before and 1.68 since the IfSG amendment. Graphically there is little change in the chosen data quality parameters (Fig 3). After the IfSG amendment there was temporarily a slight increase in the number of version of each individual case.

## Discussion

We could show a significant improvement in the timeliness of local reporting time after the IfSG amendment, without a relevant loss of data quality or identifiable increase in workload.

### Limitations

About 1/6 of all cases lack data on the local reporting time and about 1/10 of all cases lack data on the state reporting time. Local public health agencies that do not record the date of notification may have different working methods than local public health agencies that do record, which would result in a distorted local reporting time. We believe this error to be small. The missing information of the state reporting time is mostly due to the inability of the software to record that date which is not very likely to be associated with working methods to report information.

The date of the notification may be incorrectly entered by the local public health agency, because not all notifications carry a correct date of notification in them.

In contrast to other studies done on notification or reporting time we deleted cases with a delay longer than 183 days (6 months). We assume that most of those long delays come from data entry errors. Because the date of the database query was 183 days after the analysed time period, we do not assume to have a differential bias.

### Comparison with other countries

On the one hand, surveillance systems are very different across countries and change over the time, so a direct comparison is often not reasonable, on the other hand most surveillance systems aim to detect outbreaks, thus a fast reporting time should be a crucial component in all systems.

In France reporting time has been analysed by Jones et al. This study found a median delay of 6 days (Interquartile range: 4–9) in the French surveillance system between sample reception at the National Reference Centre for *Salmonella* and the availability of that information at the French Institute for Public Health [10]. Samoff et al. analysed reporting time in North Carolina, USA, and found a delay of 7 days between the initial notification to public health and the reporting to the state public health agency (electronic system in the time period January–March 2012) and 11 days for the subsequent reporting to the Centers for Disease Control and Prevention [11]. Yoo et al. published a median reporting time of 1–2 days (depending on the disease) in Korea from the local level to the state level and 0–1 days from the state level to the national level [12].

With technological advancement and the possibility of introducing or improving electronic surveillance systems the reporting time is likely going to get faster across the world.

### Amendment of the IfSG

The IfSG amendment is associated with a faster reporting time. The shift from weekly to daily reporting on the local level is clearly visible in the analysis of reporting time per weekday. The improvement of reporting time came shortly after the IfSG amendment and is consistent across local public health agencies, diseases and other factors.

The mean notification to processing time already decreased before the IfSG amendment. The subtle change by the IfSG amendment did not show a further decrease.

The parameters used to assess data quality and especially the mean number of versions as a proxy for the workload only represent selected aspects of the true data quality and workload, but the absence of a relevant change in these parameters suggest, that we have neither a relevant loss of quality nor a bigger workload for the local public health agencies. Further studies addressing the quality and workload could elucidate this point.

The IfSG amendment had the desired effect and we assume that the preconditions for early identification and management of widespread outbreaks have improved. This rapid change of practice shows that the local and state public health agencies adapt fast to new legislations. Changes in legislation are most likely a common method to improve surveillance systems. Publications on evaluations of changes in legislation are scarce [11, 13].

State public health agencies report nearly every case within one working day. We measured that the local public health agencies report two-thirds of their cases within one working day. This is limited by the fact that the reporting time of the local public health agency cannot be measured with complete accuracy, because there is no date available in the database that specifies when the case definition is met, which would require the case to be reported. There is no routine for the handling of non-compliance with the law and it is doubtful whether such a routine would be helpful for the surveillance system.

There is a large variability between the different local public health agencies. This may be due to the heterogeneous organizational structures of the different local public health agencies. An additional study investigating the knowledge, attitude and practice of the local public health agencies together with an assessment of their resources could explore reasons for slow reporting. Moreover a collection of best practice examples with continued education of local public health staff could harmonise work practices and assist local public health agencies to shorten their reporting time. The planned introduction of an electronic notification system (from physicians, laboratories and others to the local public health agency) could decrease the time needed to manually enter data in the electronic system [14]. This would likely decrease reporting time. Other studies report an improved reporting time after the introduction of an electronic notification system [11].

There is very little variability between the state public health agencies in terms of reporting time. We see limited potential to improve the state reporting time, as it is already minimal.

### Differences amongst diseases

The necessity for a fast reporting varies for the different diseases. A disease that has limited potential to cause widespread outbreaks does not need to be reported as fast as possible, other diseases including VTEC disease have a higher level of urgency. In some countries diseases are grouped into categories which have different notification times [12]. In Germany no difference is made between the statutory reporting times for different diseases, but substantial differences in the local reporting time between different diseases can be identified. Some of this variability is due to the limitation that a notification should only be reported if a case definition is fulfilled. For some diseases, for example Hepatitis B, obtaining all necessary information to decide whether the case definition is fulfilled can be time consuming. Other differences may be due to organizational structures that differ between diseases. For example reporting of tuberculosis cases are, at least in some local public health agencies, managed by tuberculosis experts, not persons usually responsible to manage notifications. Diseases with previously important widespread outbreaks as VTEC disease, Salmonellosis, Norovirus and Influenza have a median local reporting time of 1 to 2 days.

### Differences between software products

There are differences in the reporting time between software products. The results of the analysis differ between the univariable and multivariable analysis indicating a confounding by other factors that are associated with the choice of the software system. The reason for the relatively big difference is not clear, some may be due to unknown confounding, but a part might also be due to the usability of the user interface. The arrangement of the input fields for dates, differences in the description of the input fields or differences in the software manuals may change the way the user interacts with the software, thus influencing reporting time. These differences in reporting time indicate that other information in the surveillance data might also be affected by different software products. We recommend efforts to harmonise practices of local public health agencies including the exclusive use of software with fully compatible interfaces.

Software products that report cases according to specifications version 3 are associated with longer reporting time. The reason is not clear. As software specifications version 3 were introduced in 2011 and several local public health agencies implemented the new specifications during the analysed period, the longer reporting time may be due to organizational adjustments that the local public health agencies needed to implement after the update of the software. Alternatively reporting according to specifications version 3 may be more complex and thus more time consuming.

## Conclusion

The IfSG amendment from 29/03/2013 substantially decreased the reporting time from the local public health agency to the state public health agency. After the IfSG amendment the median reporting time from the date of notification to the export to the state public health agency is 1 day. The reporting time from the state public health agency to the national public health agency decreased only slightly after the IfSG amendment. The reporting time depends on the disease, on the local public health agency, on the weekday and the software used; it is independent from sex or age of the individual case.

About 40 percent of cases from local public health agencies are not reported within one working day. We recommend efforts to harmonise practices of local public health agencies including the exclusive use of software with fully compatible interfaces.

## Supporting information

S1 File R-script. (RMD)

S2 File Results of the R-Script. (HTML)
