# Supplementary material for: Timeliness in the German surveillance system for infectious diseases: Amendment of the infection protection act in 2013 decreased local reporting time to 1 day
Source: PLoS One. 2017 Oct 31;12(10):e0187037. doi: 10.1371/journal.pone.0187037 (PMC5663434; doi:10.1371/journal.pone.0187037)
Supplement: S2 File — (HTML) [file pone.0187037.s002.html]

Timeliness in the German surveillance system for infectious diseases: Amendment of the infection protection act in 2013 decreased local reporting time to 1 day.


# Timeliness in the German surveillance system for infectious diseases: Amendment of the infection protection act in 2013 decreased local reporting time to 1 day.

#### *Jakob Schumacher, Michaela Diercke, Maelle Salmon, Irina Czogiel, Claus Herrmann, Andreas Gilsdorf*

#### *22. Januar 2016*

This document calculates results for the manuscript: “Timeliness in the German surveillance system for infectious diseases: Amendment of the infection protection act in 2013 decreased local reporting time to 1 day.” Its aim is to analyze the reporting delay in the German Surveillance System. This document is the result of a statistics program called R, that can be downloaded from http://www.r-project.org.

The document is structured in three parts.

- Part one shows how the get the raw data and how data is prepared. Data is available from the Institutional Data Access of the Robert Koch Institute, Seestr. 10, 13353 Berlin for researches who meet the criteria for access to confidentioal data. See http://www.rki.de for details.
- Part two shows the analysis for each part of the manuscript.
- Part three shows background information on settings, systems and packages.

# 1 Part - Getting and preparing data

## 1.1 Querying database

The data is retrieved from the database for notifiable incetious dieases of the Robert Koch Institute. The full query can be seen at the end of the script.

## 1.2 Loading data from intermediate result and start protocol

```
load(file="rawData.RData")

# Initialize variable for protocol
protocol <- list(TotalNumberRetrieved=nrow(data))
```

## 1.3 Loading a table with disease names and other information

```
DiseaseDF <- read.table(file="Diseases.csv", header=TRUE, sep="\t", quote="", row.names = NULL, colClasses = "character", encoding="UTF-8")

datatable(rownames = FALSE, extensions = 'Buttons', DiseaseDF)
```

## 1.4 Loading a table with names of the German federal states

```
FederalstatesDF <- read.table("FederalStates.csv", header=TRUE, sep=";", row.names = NULL, colClasses = "character", encoding="UTF-8")

FederalstatesDF <- FederalstatesDF %>% 
  mutate(ReportingStateName = gsub("\xfc", "ü", ReportingStateName))

datatable(rownames = FALSE, extensions = 'Buttons',FederalstatesDF)
```

## 1.5 Loading a table with nationwide public holidays and calculate working days

```
Holidays <- read.table("Holidays.csv", header=FALSE, sep=";", row.names = NULL, colClasses = "character") %>% .[["V1"]]
WorkingDays <- sum(isBizday(timeDate(seq(as.Date("2012/03/29"), as.Date("2014/03/28"), "days")), holidays=Holidays, wday=1:5))

datatable(rownames = FALSE, extensions = 'Buttons',data.frame(Holidays))
```

## 1.6 Changing class and other minor changes

```
data <- data %>% 
  mutate_each(funs(as.Date(., format = "%Y-%m-%d")), 1:23) %>%
  mutate_each(funs(as.character(.)), 24:32,35:36) %>%
  mutate_each(funs(as.numeric(.)), 33:34, 37:40) %>%
  mutate_each(funs(replace(., .=="-nicht erhoben-", NA)), 24:32,35:36) %>% 
  mutate_each(funs(replace(., .=="ohne", NA)), 24:32,35:36) %>%
  mutate_each(funs(replace(., .=="zOther", NA)), 24:32,35:36) %>% 
  mutate_each(funs(replace(., .=="-kein-", NA)), 24:32,35:36) %>% 
  mutate(Alter = ifelse(Alter<0|Alter>120, NA, Alter)) %>% 
  mutate(SoftwareGruppeGA = factor(SoftwareGruppeGA, labels=c("Software A", "Software B", "Software C", "Software D", "SurvNet", "Software E"))) 

# Change German umlaute
levels(data$Disease) <- gsub("\xe4", "ä", levels(data$Disease)) 
levels(data$Disease) <- gsub("\xf6", "ö", levels(data$Disease)) 
levels(data$ReportingStateName) <- gsub("\xfc", "ü", levels(data$ReportingStateName))
```

## 1.7 Attach federal states to the main data

```
data <- data %>% 
  left_join(FederalstatesDF, by="ReportingStateName") %>% 
  mutate(ReportingStateName=factor(FederalStateEnglish)) %>% 
  select(-FederalStateEnglish)
```

## 1.8 Attach diseases to the main data

```
data <- data %>% 
  left_join(DiseaseDF,by="Disease") %>% 
  mutate(Erkrankung=factor(Disease)) %>% 
  mutate(Disease=factor(DiseaseEnglish)) %>% 
  select(-DiseaseEnglish)
```

## 1.9 Filter diseases introduced in IfSG amendment from 29.03.2013

```
# Protocol
protocol$DownloadedDiseases <- levels(data$Erkrankung)

"%nin%" <- function (x, table) match(x, table, nomatch = 0L) == 0L #helper function

# Filter process
data <- data %>% 
  filter(!is.na(Disease)) %>% 
  filter(Erkrankung %nin% c("Mumps", "Röteln", "Windpocken", "Keuchhusten")) %>% 
  droplevels

# Protocol
protocol$AnalysedDiseases <- levels(data$Erkrankung)
protocol$NCorrectDiseaseCategory = nrow(data)
```

## 1.10 Compute which diseases are frequent and filter those with less than 10 cases

```
data <- data %>% 
  left_join(data %>% 
              count(Disease) %>% 
              filter(n>10) %>% 
              mutate(FrequentDisease= TRUE) %>% 
              select(Disease, FrequentDisease), by="Disease")

# Filter
data <- data %>% filter(FrequentDisease)

# Protocol
protocol$TotalNumber = nrow(data)
protocol$FilterLess10Cases = protocol$NCorrectDiseaseCategory-protocol$TotalNumber
```

## 1.11 Find the first date of notification

```
data <- data %>% 
    mutate(dateOfNotification = as.Date(pmin(ArztMeldungImGA, LaborMeldungImGA, EigeneErmittlungMeldungImGA, GemeinschaftP8MeldungImGA, WeiterleitungAnderesGAMeldungImGA, GemeinschaftP34MeldungImGA, AndereMeldungImGA, UnbekannteMeldeArtImGA, na.rm=TRUE), origin="1970-01-01"))
```

## 1.12 Compute which days are weekdays

```
data <- data %>%
  mutate(Weekday_local = weekdays(dateOfNotification)) %>%
  mutate(Weekday_local = factor(Weekday_local, levels=c("Montag", "Dienstag", "Mittwoch", "Donnerstag", "Freitag", "Samstag", "Sonntag"), labels=c("Monday", "Tuesday", "Wednesday", "Thursday", "Friday", "Saturday", "Sunday"))) %>%
  mutate(Weekday_state = weekdays(ImportLS)) %>%
  mutate(Weekday_state = factor(Weekday_state, levels=c("Montag", "Dienstag", "Mittwoch", "Donnerstag", "Freitag", "Samstag", "Sonntag"), labels=c("Monday", "Tuesday", "Wednesday", "Thursday", "Friday", "Saturday", "Sunday")))
```

## 1.13 Categorise data in cases before and after the IfSG amendment

```
data <- data %>% 
  mutate(gesetz_local = factor(ifelse(Meldedatum <= as.Date("2013-03-31"), "VorGesetz", "NachGesetz"))) %>%
  mutate(gesetz_state = factor(ifelse(ImportLS <= as.Date("2013-03-31"), "VorGesetz", "NachGesetz")))
```

## 1.14 Find local public health agencies that have reported more than 10 cases according to specifications version 3 before the IfSG amendment

```
data <- data %>% 
  left_join(data %>% 
              filter(SoftwareSN2SN3GA=="SN3") %>% 
              filter(gesetz_local=="VorGesetz") %>% 
              count(MeldendesGA) %>% 
              filter(n>10) %>% 
              mutate(longtermSN3User= TRUE) %>% 
              select(MeldendesGA, longtermSN3User), by="MeldendesGA")
```

## 1.15 Compute delays

```
data <- data %>% 
  mutate(delay_to_report_local = as.numeric(ExportGA - dateOfNotification)) %>%
  mutate(delay_to_report_state = as.numeric(ExportLS - ImportLS)) %>% 
  mutate(notificationProcessDelay = as.numeric(Meldedatum-dateOfNotification)) %>% 
  mutate(total_delay = as.numeric(ExportLS-dateOfNotification))
```

## 1.16 Protocol

```
protocol$dateOfNotification=sum(!is.na(data$dateOfNotification))
protocol$NLocalPublicHealthAgencies=length(levels(as.factor(data$MeldendesGA)))
protocol$ExportGA=sum(!is.na(data$ExportGA))
protocol$ImportLS=sum(!is.na(data$ImportLS)) 
protocol$ExportLS=sum(!is.na(data$ExportLS)) 
protocol$DelayLocalInitial=sum(!is.na(data$delay_to_report_local))
protocol$DelayStateInitial=sum(!is.na(data$delay_to_report_state))
protocol$DelayLocalUnder0=sum(data$delay_to_report_local<0, na.rm=TRUE)
protocol$DelayStateUnder0=sum(data$delay_to_report_state<0, na.rm=TRUE)
protocol$DelayLocalAbove183=sum(data$delay_to_report_local>183, na.rm=TRUE)
protocol$DelayStateAbove183=sum(data$delay_to_report_state>183, na.rm=TRUE)
```

## 1.17 Deleting delay values that are potentially wrong

```
data <- data %>% 
     mutate(delay_to_report_local = ifelse(delay_to_report_local<0|delay_to_report_local>183, NA, delay_to_report_local))%>%
     mutate(delay_to_report_state = ifelse(delay_to_report_state<0|delay_to_report_state>183, NA, delay_to_report_state))%>%
     mutate(notificationProcessDelay = ifelse(notificationProcessDelay<0|notificationProcessDelay>183, NA, notificationProcessDelay)) %>% 
  mutate(total_delay = ifelse(total_delay<0|total_delay>183, NA, total_delay)) 

# Deleting delay values of processing delay that dont fall on a Mo-Thu and only those with SN3
data <- data %>% 
  mutate(notificationProcessDelay = ifelse(Weekday_local %in% c("Friday", "Saturday", "Sunday"), NA, notificationProcessDelay)) %>% 
  mutate(notificationProcessDelay = ifelse(SoftwareSN2SN3GA=="SN2", NA, notificationProcessDelay)) %>% 
  mutate(notificationProcessDelay = ifelse(!longtermSN3User, NA, notificationProcessDelay))
```

## 1.18 Categorising into quartiles

```
data <- data %>% 
  left_join(data %>% 
              count(MeldendesGA) %>% 
              mutate(NotificationsPerWorkingDay = n/WorkingDays) %>% 
              mutate(rangNotifications= ntile(NotificationsPerWorkingDay,4)) ,by="MeldendesGA")

# We label the rangs according to meanNotificationsPerWorkingDay
Label1 <- round(min(data$NotificationsPerWorkingDay[data$rangNotifications==1], na.rm=T),1)
Label2 <- round(min(data$NotificationsPerWorkingDay[data$rangNotifications==2], na.rm=T),1)
Label3 <- round(min(data$NotificationsPerWorkingDay[data$rangNotifications==3], na.rm=T),1)
Label4 <- round(min(data$NotificationsPerWorkingDay[data$rangNotifications==4], na.rm=T),1)
Label5 <- round(max(data$NotificationsPerWorkingDay[data$rangNotifications==4], na.rm=T),1)
AllLabels <- c(paste(Label1, Label2, sep=" - "), 
               paste(Label2, Label3, sep=" - "), 
               paste(Label3, Label4, sep=" - "), 
               paste(Label4, Label5, sep=" - "))

data$rangNotifications <- factor(data$rangNotifications, labels=AllLabels)
rm(Label1, Label2, Label3, Label4, Label5, AllLabels)

# Introduce rangTransmission
data <- data %>% 
  left_join(data %>%
              filter(gesetz_local=="VorGesetz") %>% 
              group_by(MeldendesGA) %>% 
              summarise(mean = mean(delay_to_report_local, na.rm=TRUE)) %>% 
              mutate(rangTransmission= ntile(mean,4)) %>%
              select(MeldendesGA, rangTransmission) 
            ,by="MeldendesGA")
```

## 1.19 Compute agegroup

```
data <- data %>%
  mutate(Agegroup = findInterval(Alter, c(seq(0,100,10)))) %>% 
  mutate(Agegroup = ifelse(Agegroup>10, 10, Agegroup)) %>% 
  mutate(Agegroup = factor(Agegroup, labels=c("0-9", "10-19", "20-29", "30-39", "40-49", "50-59", "60-69", "70-79", "80-89", "90+")))
```

## 1.20 Compute a variable for the labeling of the figures

```
data <- data %>% mutate(Yearmonth = as.Date(paste(strftime(Meldedatum, format="%Y-%m"), "01", sep="-")))
```

## 1.21 Find the local public health agencies that report within one working day

```
data$wd0 <- isBizday(timeDate(data$dateOfNotification), holidays=Holidays, wday=1:5)
data$wd1 <- isBizday(timeDate(data$dateOfNotification+1), holidays=Holidays, wday=1:5)
data$wd2 <- isBizday(timeDate(data$dateOfNotification+2), holidays=Holidays, wday=1:5)
data$wd3 <- isBizday(timeDate(data$dateOfNotification+3), holidays=Holidays, wday=1:5)
data$wd4 <- isBizday(timeDate(data$dateOfNotification+4), holidays=Holidays, wday=1:5)
data$wd5 <- isBizday(timeDate(data$dateOfNotification+5), holidays=Holidays, wday=1:5)
data$wd6 <- isBizday(timeDate(data$dateOfNotification+6), holidays=Holidays, wday=1:5)

data<-data %>% 
    mutate(target=0) %>% 
    mutate(target=ifelse(wd0 & wd6, 6,target) ) %>% 
    mutate(target=ifelse(wd0 & wd5, 5,target) ) %>% 
    mutate(target=ifelse(wd0 & wd4, 4,target) ) %>% 
    mutate(target=ifelse(wd0 & wd3, 3,target) ) %>% 
    mutate(target=ifelse(wd0 & wd2, 2,target) ) %>% 
    mutate(target=ifelse(wd0 & wd1, 1,target) ) %>% 
    mutate(target=ifelse(!wd0 & wd1 & wd6, 6,target) ) %>% 
    mutate(target=ifelse(!wd0 & wd1 & wd5, 5,target) ) %>% 
    mutate(target=ifelse(!wd0 & wd1 & wd4, 4,target) ) %>% 
    mutate(target=ifelse(!wd0 & wd1 & wd3, 3,target) ) %>%
    mutate(target=ifelse(!wd0 & wd1 & wd2, 2,target) ) %>%
    mutate(target=ifelse(!wd0 & !wd1 & wd2 & wd6, 6,target) ) %>% 
    mutate(target=ifelse(!wd0 & !wd1 & wd2 & wd5, 5,target) ) %>% 
    mutate(target=ifelse(!wd0 & !wd1 & wd2 & wd4, 4,target) ) %>% 
    mutate(target=ifelse(!wd0 & !wd1 & wd2 & wd3, 3,target) ) %>%
    mutate(target=ifelse(!wd0 & !wd1 & !wd2 & wd3 & wd6, 6,target) ) %>% 
    mutate(target=ifelse(!wd0 & !wd1 & !wd2 & wd3 & wd5, 5,target) ) %>% 
    mutate(target=ifelse(!wd0 & !wd1 & !wd2 & wd3 & wd4, 4,target) ) %>% 
    mutate(target=ifelse(!wd0 & !wd1 & !wd2 & !wd3 & wd4 & wd6, 6,target) ) %>% 
    mutate(target=ifelse(!wd0 & !wd1 & !wd2 & !wd3 & wd4 & wd5, 5,target) ) %>% 
    mutate(target=ifelse(!wd0 & !wd1 & !wd2 & !wd3 & !wd4 & wd5 & wd6, 6 ,target) ) %>%
    mutate(target=ifelse(!wd0 & !wd1 & !wd2 & !wd3 & !wd4 & !wd5 & !wd6, NA ,target) ) 

# correct transmission
data <- data %>% 
    mutate(korrekt_local = delay_to_report_local - target) %>% 
    mutate(korrekt_local = factor(ifelse(korrekt_local<=0,1,2), labels=c("fullfilled", "notfullfilled")))
```

## 1.22 Find the state public health agencies that report within one working day

```
data$wd0 <- isBizday(timeDate(data$ImportLS), holidays=Holidays, wday=1:5)
data$wd1 <- isBizday(timeDate(data$ImportLS+1), holidays=Holidays, wday=1:5)
data$wd2 <- isBizday(timeDate(data$ImportLS+2), holidays=Holidays, wday=1:5)
data$wd3 <- isBizday(timeDate(data$ImportLS+3), holidays=Holidays, wday=1:5)
data$wd4 <- isBizday(timeDate(data$ImportLS+4), holidays=Holidays, wday=1:5)
data$wd5 <- isBizday(timeDate(data$ImportLS+5), holidays=Holidays, wday=1:5)
data$wd6 <- isBizday(timeDate(data$ImportLS+6), holidays=Holidays, wday=1:5)

data<-data %>% 
    mutate(target=0) %>% 
    mutate(target=ifelse(wd0 & wd6, 6,target) ) %>% 
    mutate(target=ifelse(wd0 & wd5, 5,target) ) %>% 
    mutate(target=ifelse(wd0 & wd4, 4,target) ) %>% 
    mutate(target=ifelse(wd0 & wd3, 3,target) ) %>% 
    mutate(target=ifelse(wd0 & wd2, 2,target) ) %>% 
    mutate(target=ifelse(wd0 & wd1, 1,target) ) %>% 
    mutate(target=ifelse(!wd0 & wd1 & wd6, 6,target) ) %>% 
    mutate(target=ifelse(!wd0 & wd1 & wd5, 5,target) ) %>% 
    mutate(target=ifelse(!wd0 & wd1 & wd4, 4,target) ) %>% 
    mutate(target=ifelse(!wd0 & wd1 & wd3, 3,target) ) %>%
    mutate(target=ifelse(!wd0 & wd1 & wd2, 2,target) ) %>%
    mutate(target=ifelse(!wd0 & !wd1 & wd2 & wd6, 6,target) ) %>% 
    mutate(target=ifelse(!wd0 & !wd1 & wd2 & wd5, 5,target) ) %>% 
    mutate(target=ifelse(!wd0 & !wd1 & wd2 & wd4, 4,target) ) %>% 
    mutate(target=ifelse(!wd0 & !wd1 & wd2 & wd3, 3,target) ) %>%
    mutate(target=ifelse(!wd0 & !wd1 & !wd2 & wd3 & wd6, 6,target) ) %>% 
    mutate(target=ifelse(!wd0 & !wd1 & !wd2 & wd3 & wd5, 5,target) ) %>% 
    mutate(target=ifelse(!wd0 & !wd1 & !wd2 & wd3 & wd4, 4,target) ) %>% 
    mutate(target=ifelse(!wd0 & !wd1 & !wd2 & !wd3 & wd4 & wd6, 6,target) ) %>% 
    mutate(target=ifelse(!wd0 & !wd1 & !wd2 & !wd3 & wd4 & wd5, 5,target) ) %>% 
    mutate(target=ifelse(!wd0 & !wd1 & !wd2 & !wd3 & !wd4 & wd5 & wd6, 6 ,target) ) %>%
    mutate(target=ifelse(!wd0 & !wd1 & !wd2 & !wd3 & !wd4 & !wd5 & !wd6, NA ,target) ) 

# correct transmission
data <- data %>% 
    mutate(korrekt_state = delay_to_report_state - target) %>% 
    mutate(korrekt_state = factor(ifelse(korrekt_state<=0,1,2), labels=c("fullfilled", "notfullfilled")))
```

# 2 Part - Results

## 2.1 Data selection

$TotalNumber [1] 665401

$dateOfNotification [1] 621869

$NLocalPublicHealthAgencies [1] 392

$ExportGA [1] 590337

$ImportLS [1] 606055

$ExportLS [1] 665375

$DelayLocalInitial [1] 550095

$DelayStateInitial [1] 606029

$DelayLocalUnder0 [1] 401

$DelayStateUnder0 [1] 1382

$DelayLocalAbove183 [1] 1810

$DelayStateAbove183 [1] 19

## 2.2 Analysis of local reporting time

```
 Univariable_Overall <- data %>%
  filter(!is.na(delay_to_report_local)) %>% 
  group_by(gesetz_local) %>%
  summarise(
    median=quantile(delay_to_report_local, probs = c(0.5), na.rm=TRUE),
    q25 = quantile(delay_to_report_local, probs = c(0.25), na.rm=TRUE),
    q75 = quantile(delay_to_report_local, probs = c(0.75), na.rm=TRUE),
    n=n()) %>% 
  mutate(median = paste0(n, "_", median, " (", q25, " - ", q75,")")) %>% 
  select(gesetz_local, median) %>% 
  spread(key=gesetz_local, value=median) %>%
  separate(VorGesetz, into=c("n_before", "median_before"), sep="_") %>% 
  separate(NachGesetz, into=c("n_after", "median_after"), sep="_") %>%
  mutate(n_before=as.numeric(n_before)) %>% 
  mutate(p_before=round(n_before*100/sum(n_before))) %>%
  mutate(n_before = paste0(n_before, "(", p_before, ")")) %>% 
  mutate(n_after=as.numeric(n_after)) %>% 
  mutate(p_after=round(n_after*100/sum(n_after))) %>% 
  mutate(n_after = paste0(n_after, "(", p_after, ")")) %>% 
  mutate(Variable = "Overall") %>% 
  select(Variable, n_before, median_before, n_after, median_after)  


Univariable_GA<- data %>%
  filter(!is.na(rangTransmission)) %>% 
  filter(!is.na(delay_to_report_local)) %>% 
  group_by(gesetz_local, rangTransmission) %>%
  summarise(
    median=quantile(delay_to_report_local, probs = c(0.5), na.rm=TRUE),
    q25 = quantile(delay_to_report_local, probs = c(0.25), na.rm=TRUE),
    q75 = quantile(delay_to_report_local, probs = c(0.75), na.rm=TRUE),
    n=n()) %>% 
  mutate(median = paste0(n, "_", median, " (", q25, " - ", q75,")")) %>% 
  select(gesetz_local, rangTransmission, median) %>% 
  spread(key=gesetz_local, value=median) %>%
  separate(VorGesetz, into=c("n_before", "median_before"), sep="_") %>% 
  separate(NachGesetz, into=c("n_after", "median_after"), sep="_") %>%
  mutate(n_before=as.numeric(n_before)) %>% 
  mutate(p_before=round(n_before*100/sum(n_before))) %>%
  mutate(n_before = paste0(n_before, "(", p_before, ")")) %>% 
  mutate(n_after=as.numeric(n_after)) %>% 
  mutate(p_after=round(n_after*100/sum(n_after))) %>% 
  mutate(n_after = paste0(n_after, "(", p_after, ")")) %>% 
  mutate(rangTransmission = factor(rangTransmission, levels=c(1,2,3,4), labels=c("Local public health agency - fastes quarter", "Local public health agency - second fastest quarter", "Local public health agency - third fastest quarter", "Local public health agency - slowest quarter"))) %>%
  select(Variable = rangTransmission, n_before, median_before, n_after, median_after)  

Univariable_DayOfWeek <- data %>%
  filter(!is.na(Weekday_local)) %>% 
  filter(!is.na(delay_to_report_local)) %>% 
  group_by(gesetz_local, Weekday_local) %>%
  summarise(
    median=quantile(delay_to_report_local, probs = c(0.5), na.rm=TRUE),
    q25 = quantile(delay_to_report_local, probs = c(0.25), na.rm=TRUE),
    q75 = quantile(delay_to_report_local, probs = c(0.75), na.rm=TRUE),
    n=n()) %>% 
  mutate(median = paste0(n, "_", median, " (", q25, " - ", q75,")")) %>% 
  select(gesetz_local, Weekday_local, median) %>% 
  spread(key=gesetz_local, value=median) %>%
  separate(VorGesetz, into=c("n_before", "median_before"), sep="_") %>% 
  separate(NachGesetz, into=c("n_after", "median_after"), sep="_") %>%
  mutate(n_before=as.numeric(n_before)) %>% 
  mutate(p_before=round(n_before*100/sum(n_before))) %>%
  mutate(n_before = paste0(n_before, "(", p_before, ")")) %>% 
  mutate(n_after=as.numeric(n_after)) %>% 
  mutate(p_after=round(n_after*100/sum(n_after))) %>% 
  mutate(n_after = paste0(n_after, "(", p_after, ")")) %>% 
  select(Variable = Weekday_local, n_before, median_before, n_after, median_after)  

Univariable_Software <- data %>%
  filter(!is.na(SoftwareGruppeGA)) %>% 
  filter(!is.na(delay_to_report_local)) %>% 
  group_by(gesetz_local, SoftwareGruppeGA) %>%
  summarise(
    median=quantile(delay_to_report_local, probs = c(0.5), na.rm=TRUE),
    q25 = quantile(delay_to_report_local, probs = c(0.25), na.rm=TRUE),
    q75 = quantile(delay_to_report_local, probs = c(0.75), na.rm=TRUE),
    n=n()) %>% 
  mutate(median = paste0(n, "_", median, " (", q25, " - ", q75,")")) %>% 
  select(gesetz_local, SoftwareGruppeGA, median) %>% 
  spread(key=gesetz_local, value=median) %>%
  separate(VorGesetz, into=c("n_before", "median_before"), sep="_") %>% 
  separate(NachGesetz, into=c("n_after", "median_after"), sep="_") %>%
  mutate(n_before=as.numeric(n_before)) %>% 
  mutate(p_before=round(n_before*100/sum(n_before))) %>%
  mutate(n_before = paste0(n_before, "(", p_before, ")")) %>% 
  mutate(n_after=as.numeric(n_after)) %>% 
  mutate(p_after=round(n_after*100/sum(n_after))) %>% 
  mutate(n_after = paste0(n_after, "(", p_after, ")")) %>% 
  select(Variable = SoftwareGruppeGA, n_before, median_before, n_after, median_after)

Univariable_SoftwareSN2SN3GA <- data %>%
  filter(!is.na(SoftwareSN2SN3GA)) %>% 
  filter(!is.na(delay_to_report_local)) %>% 
  group_by(gesetz_local, SoftwareSN2SN3GA) %>%
  summarise(
    median=quantile(delay_to_report_local, probs = c(0.5), na.rm=TRUE),
    q25 = quantile(delay_to_report_local, probs = c(0.25), na.rm=TRUE),
    q75 = quantile(delay_to_report_local, probs = c(0.75), na.rm=TRUE),
    n=n()) %>% 
  mutate(median = paste0(n, "_", median, " (", q25, " - ", q75,")")) %>% 
  select(gesetz_local, SoftwareSN2SN3GA, median) %>% 
  spread(key=gesetz_local, value=median) %>%
  separate(VorGesetz, into=c("n_before", "median_before"), sep="_") %>% 
  separate(NachGesetz, into=c("n_after", "median_after"), sep="_") %>%
  mutate(n_before=as.numeric(n_before)) %>% 
  mutate(p_before=round(n_before*100/sum(n_before))) %>%
  mutate(n_before = paste0(n_before, "(", p_before, ")")) %>% 
  mutate(n_after=as.numeric(n_after)) %>% 
  mutate(p_after=round(n_after*100/sum(n_after))) %>% 
  mutate(n_after = paste0(n_after, "(", p_after, ")")) %>% 
  select(Variable = SoftwareSN2SN3GA, n_before, median_before, n_after, median_after)  

Univariable_NotificationsPerDay <- data %>%
  filter(!is.na(rangNotifications)) %>% 
  filter(!is.na(delay_to_report_local)) %>% 
  group_by(gesetz_local, rangNotifications) %>%
  summarise(
    median=quantile(delay_to_report_local, probs = c(0.5), na.rm=TRUE),
    q25 = quantile(delay_to_report_local, probs = c(0.25), na.rm=TRUE),
    q75 = quantile(delay_to_report_local, probs = c(0.75), na.rm=TRUE),
    n=n()) %>% 
  mutate(median = paste0(n, "_", median, " (", q25, " - ", q75,")")) %>% 
  select(gesetz_local, rangNotifications, median) %>% 
  spread(key=gesetz_local, value=median) %>%
  separate(VorGesetz, into=c("n_before", "median_before"), sep="_") %>% 
  separate(NachGesetz, into=c("n_after", "median_after"), sep="_") %>%
  mutate(n_before=as.numeric(n_before)) %>% 
  mutate(p_before=round(n_before*100/sum(n_before))) %>%
  mutate(n_before = paste0(n_before, "(", p_before, ")")) %>% 
  mutate(n_after=as.numeric(n_after)) %>% 
  mutate(p_after=round(n_after*100/sum(n_after))) %>% 
  mutate(n_after = paste0(n_after, "(", p_after, ")")) %>% 
  select(Variable = rangNotifications, n_before, median_before, n_after, median_after)

EmptyLine <- data.frame(Variable=" ", n_before=" ", median_before=" ", n_after=" ", median_after=" ")

TableLocal <- rbind(Univariable_Overall, EmptyLine, 
                    Univariable_GA, EmptyLine,
                    Univariable_Software, EmptyLine, 
                    Univariable_SoftwareSN2SN3GA, EmptyLine, 
                    Univariable_DayOfWeek, EmptyLine,
                    Univariable_NotificationsPerDay)

rm(Univariable_Overall,Univariable_GA, Univariable_Software, Univariable_SoftwareSN2SN3GA, Univariable_DayOfWeek, Univariable_NotificationsPerDay)
```

```
# Multivariable model
if (!require("MASS")) install.packages('MASS'); require("MASS")
select <- dplyr::select
data$SoftwareGruppeGA <- relevel(data$SoftwareGruppeGA, ref = "SurvNet")
data$Disease <- relevel(data$Disease, ref = "norovirus gastroenteritis")

formula <- delay_to_report_local ~  
  Weekday_local +
  gesetz_local +                          
  rangNotifications +
  Disease +
  SoftwareGruppeGA +
  SoftwareSN2SN3GA 

BinregModel<- glm.nb(formula = formula, data=data)

BinregDF<-data.frame(coef(summary(BinregModel))) %>% 
  add_rownames() %>% 
  mutate(Exp = round(exp(Estimate),2)) %>%
  mutate(expLCI = round(exp(Estimate-1.96*Std..Error),2)) %>% 
  mutate(expUCI = round(exp(Estimate+1.96*Std..Error),2)) %>% 
  mutate(CI = paste(expLCI, expUCI, sep="-")) %>% 
  rename(p=Pr...z.., Variable=rowname) %>% 
  mutate(p = round(p,3)) %>% 
  filter(!grepl("Disease", Variable)) %>% 
  mutate(Variable=ifelse(grepl("Weekday_local", Variable), substring(Variable, 14), Variable)) %>%
  mutate(Variable=ifelse(grepl("rangNotifications", Variable), substring(Variable, 18), Variable)) %>% 
  mutate(Variable=ifelse(grepl("SoftwareGruppeGA", Variable), substring(Variable, 17), Variable)) %>%
  mutate(Variable=ifelse(grepl("SoftwareSN2SN3GA", Variable), substring(Variable, 17), Variable)) %>%
  mutate(Variable=ifelse(grepl("gesetz_local", Variable), substring(Variable, 13), Variable)) %>%
  select(Variable, Exp, CI)

TableLocal<- TableLocal %>% left_join(BinregDF, by="Variable") 

datatable(rownames = FALSE, extensions = 'Buttons',TableLocal %>% filter(Variable=="Overall"))
```

## 2.3 Figure 2

```
plotLocal <- data %>% 
  filter(Yearmonth>=as.Date("2012-04-01")&Yearmonth<as.Date("2014-04-01")) %>% 
  select(Yearmonth, delay_to_report_local) %>% na.omit %>% 
  group_by(Yearmonth) %>% 
  summarise(n=n(),mn=mean(delay_to_report_local), sd=sd(delay_to_report_local)) %>% 
  mutate(se=sd/sqrt(n),LCI=mn+qnorm(0.025)*se,UCI=mn+qnorm(0.975)*se) %>% 
  ggplot(aes(x=Yearmonth, y=mn)) +
  geom_bar(stat="identity", fill="steelblue") +
  geom_errorbar(aes(ymin=LCI, ymax=UCI))+
  theme_classic(base_size = 14) +
  theme(axis.text.x  = element_text(angle=45, hjust=1, vjust=1))+
  geom_vline(xintercept=as.numeric(as.Date("2013-03-01")), linetype="dashed")+
  scale_x_date(date_break="2 months", date_labels="%Y-%m") +
  labs(x="Time in months", y="local reporting \n time in days")
  theme(legend.position="none",plot.margin=unit(c(0,0,0,0), "mm"))
```

```
## List of 2
##  $ legend.position: chr "none"
##  $ plot.margin    :Class 'unit'  atomic [1:4] 0 0 0 0
##   .. ..- attr(*, "valid.unit")= int 7
##   .. ..- attr(*, "unit")= chr "mm"
##  - attr(*, "class")= chr [1:2] "theme" "gg"
##  - attr(*, "complete")= logi FALSE
##  - attr(*, "validate")= logi TRUE
```

```
plotState <- data %>% 
  filter(Yearmonth>=as.Date("2012-04-01")&Yearmonth<as.Date("2014-04-01")) %>% 
  select(Yearmonth, delay_to_report_state) %>% na.omit %>% 
  group_by(Yearmonth) %>% 
  summarise(n=n(),mn=mean(delay_to_report_state), sd=sd(delay_to_report_state)) %>% 
  mutate(se=sd/sqrt(n),LCI=mn+qnorm(0.025)*se,UCI=mn+qnorm(0.975)*se) %>% 
  ggplot(aes(x=Yearmonth, y=mn)) +
  geom_bar(stat="identity", fill="steelblue") +
  geom_errorbar(aes(ymin=LCI, ymax=UCI))+
  theme_classic(base_size = 14) +
  theme(axis.text.x  = element_text(angle=45, hjust=1, vjust=1))+
  geom_vline(xintercept=as.numeric(as.Date("2013-03-01")), linetype="dashed")+
  scale_x_date(date_break="2 months", date_labels="%Y-%m") +
  scale_y_continuous(limits=c(0,8))+
  labs(x="Time in months", y="state reporting \n time in days")
  theme(legend.position="none",plot.margin=unit(c(0,0,0,0), "mm"))
```

```
## List of 2
##  $ legend.position: chr "none"
##  $ plot.margin    :Class 'unit'  atomic [1:4] 0 0 0 0
##   .. ..- attr(*, "valid.unit")= int 7
##   .. ..- attr(*, "unit")= chr "mm"
##  - attr(*, "class")= chr [1:2] "theme" "gg"
##  - attr(*, "complete")= logi FALSE
##  - attr(*, "validate")= logi TRUE
```

```
plotNP <- data %>% 
  filter(longtermSN3User) %>% 
  filter(SoftwareSN2SN3GA=="SN3") %>% 
  filter(Yearmonth>=as.Date("2012-04-01")&Yearmonth<as.Date("2014-04-01")) %>% 
  group_by(Yearmonth) %>% 
  summarise(n=n(),mn=mean(notificationProcessDelay, na.rm=T), sd=sd(notificationProcessDelay, na.rm=T)) %>% 
  mutate(se=sd/sqrt(n),LCI=mn+qnorm(0.025)*se,UCI=mn+qnorm(0.975)*se) %>% 
  ggplot(aes(x=Yearmonth, y=mn)) +
  geom_bar(stat="identity", fill="steelblue") +
  geom_errorbar(aes(ymax = UCI, ymin=LCI)) +
  theme_classic(base_size = 14) +
  theme(axis.text.x  = element_text(angle=45, hjust=1, vjust=1))+
  geom_vline(xintercept=as.numeric(as.Date("2013-03-01")), linetype="dashed")+
  scale_x_date(date_break="2 months", date_labels="%Y-%m") +
  scale_y_continuous(limits=c(0,8))+
  labs(x="Time in months", y="mean notification \n process time \n in days")
```

```
# Plotting
timeplot <- grid.arrange(plotLocal, plotState, plotNP, ncol=1)
```

```
fileheight=8
setEPS()
postscript("figure2.eps", height = fileheight, width = fileheight*0.7)
 grid.arrange(plotLocal, plotState, plotNP, ncol=1)
dev.off()
```

png 2

## 2.4 Table 1

```
datatable(rownames = FALSE, extensions = 'Buttons',TableLocal)
```

## 2.5 Differences among local public health agencies

```
datatable(rownames = FALSE, extensions = 'Buttons',TableLocal %>% filter(grepl('quarter', Variable)))
```

## 2.6 Software

```
datatable(rownames = FALSE, extensions = 'Buttons',TableLocal %>% filter(grepl('    SurvNet|Software|SN', Variable)))
```

## 2.7 Weekday

```
datatable(rownames = FALSE, extensions = 'Buttons',TableLocal %>% filter(grepl('day', Variable)))
```

## 2.8 Number of notifcations per day

```
datatable(rownames = FALSE, extensions = 'Buttons',TableLocal %>% filter(grepl('1.6|4.3', Variable)))
```

## 2.9 Age and sex

```
datatable(rownames = FALSE, extensions = 'Buttons',
data %>%
  filter(!is.na(Geschlecht)) %>% 
  filter(!is.na(delay_to_report_local)) %>% 
  group_by(gesetz_local, Geschlecht) %>%
  summarise(
    median=quantile(delay_to_report_local, probs = c(0.5), na.rm=TRUE),
    q25 = quantile(delay_to_report_local, probs = c(0.25), na.rm=TRUE),
    q75 = quantile(delay_to_report_local, probs = c(0.75), na.rm=TRUE),
    n=n()) %>% 
  mutate(median = paste0(n, "_", median, " (", q25, " - ", q75,")")) %>% 
  select(gesetz_local, Geschlecht, median) %>% 
  spread(key=gesetz_local, value=median) %>%
  separate(VorGesetz, into=c("n_before", "median_before"), sep="_") %>% 
  separate(NachGesetz, into=c("n_after", "median_after"), sep="_") %>%
  mutate(n_before=as.numeric(n_before)) %>% 
  mutate(p_before=round(n_before*100/sum(n_before))) %>%
  mutate(n_before = paste0(n_before, "(", p_before, ")")) %>% 
  mutate(n_after=as.numeric(n_after)) %>% 
  mutate(p_after=round(n_after*100/sum(n_after))) %>% 
  mutate(n_after = paste0(n_after, "(", p_after, ")")) %>% 
  select(Variable = Geschlecht, n_before, median_before, n_after, median_after)
)
```

```
datatable(rownames = FALSE, extensions = 'Buttons',
data %>%
  filter(!is.na(Agegroup)) %>% 
  filter(!is.na(delay_to_report_local)) %>% 
  group_by(gesetz_local, Agegroup) %>%
  summarise(
    median=quantile(delay_to_report_local, probs = c(0.5), na.rm=TRUE),
    q25 = quantile(delay_to_report_local, probs = c(0.25), na.rm=TRUE),
    q75 = quantile(delay_to_report_local, probs = c(0.75), na.rm=TRUE),
    n=n()) %>% 
  mutate(median = paste0(n, "_", median, " (", q25, " - ", q75,")")) %>% 
  select(gesetz_local, Agegroup, median) %>% 
  spread(key=gesetz_local, value=median) %>%
  separate(VorGesetz, into=c("n_before", "median_before"), sep="_") %>% 
  separate(NachGesetz, into=c("n_after", "median_after"), sep="_") %>%
  mutate(n_before=as.numeric(n_before)) %>% 
  mutate(p_before=round(n_before*100/sum(n_before))) %>%
  mutate(n_before = paste0(n_before, "(", p_before, ")")) %>% 
  mutate(n_after=as.numeric(n_after)) %>% 
  mutate(p_after=round(n_after*100/sum(n_after))) %>% 
  mutate(n_after = paste0(n_after, "(", p_after, ")")) %>% 
  select(Variable = Agegroup, n_before, median_before, n_after, median_after)
)
```

## 2.10 Table 2

```
BinregDiseaseDF<-data.frame(coef(summary(BinregModel))) %>% 
  add_rownames() %>% 
  mutate(Exp = round(exp(Estimate),2)) %>%
  mutate(expLCI = round(exp(Estimate-1.96*Std..Error),2)) %>% 
  mutate(expUCI = round(exp(Estimate+1.96*Std..Error),2)) %>% 
  mutate(CI = paste(expLCI, expUCI, sep="-")) %>% 
  rename(p=Pr...z.., Variable=rowname) %>% 
  mutate(p = round(p,3)) %>% 
  mutate(Variable=ifelse(grepl("Disease", Variable), substring(Variable, 8), Variable)) %>% 
  select(Variable, Exp, CI)
 

DiseasesLocal <- data %>%
  filter(!is.na(Disease)) %>% 
  filter(!is.na(delay_to_report_local)) %>% 
  group_by(gesetz_local, Disease) %>%
  summarise(
    median=quantile(delay_to_report_local, probs = c(0.5), na.rm=TRUE),
    q25 = quantile(delay_to_report_local, probs = c(0.25), na.rm=TRUE),
    q75 = quantile(delay_to_report_local, probs = c(0.75), na.rm=TRUE),
    n=n()) %>% 
  mutate(median = paste0(n, "_", median, " (", q25, " - ", q75,")")) %>% 
  select(gesetz_local, Disease, median) %>% 
  spread(key=gesetz_local, value=median) %>%
  separate(VorGesetz, into=c("n_before", "median_before"), sep="_") %>% 
  separate(NachGesetz, into=c("n_after", "median_after"), sep="_") %>%
  mutate(n_before=as.numeric(n_before)) %>% 
  mutate(p_before=round(n_before*100/sum(n_before))) %>%
  mutate(n_before = paste0(n_before, "(", p_before, ")")) %>% 
  mutate(n_after=as.numeric(n_after)) %>% 
  mutate(p_after=round(n_after*100/sum(n_after))) %>% 
  mutate(n_after = paste0(n_after, "(", p_after, ")")) %>% 
  select(Variable = Disease, n_before, median_before, n_after, median_after)  


DiseasesState <- data %>%
  filter(!is.na(Disease)) %>% 
  filter(!is.na(delay_to_report_state)) %>% 
  group_by(gesetz_state, Disease) %>%
  summarise(
    median=quantile(delay_to_report_state, probs = c(0.5), na.rm=TRUE),
    q25 = quantile(delay_to_report_state, probs = c(0.25), na.rm=TRUE),
    q75 = quantile(delay_to_report_state, probs = c(0.75), na.rm=TRUE),
    n=n()) %>% 
  mutate(median = paste0(n, "_", median, " (", q25, " - ", q75,")")) %>% 
  select(gesetz_state, Disease, median) %>% 
  spread(key=gesetz_state, value=median) %>%
  separate(VorGesetz, into=c("n_before", "median_before"), sep="_") %>% 
  separate(NachGesetz, into=c("n_after", "median_after"), sep="_") %>%
  mutate(n_before=as.numeric(n_before)) %>% 
  mutate(p_before=round(n_before*100/sum(n_before))) %>%
  mutate(n_before = paste0(n_before, "(", p_before, ")")) %>% 
  mutate(n_after=as.numeric(n_after)) %>% 
  mutate(p_after=round(n_after*100/sum(n_after))) %>% 
  mutate(n_after = paste0(n_after, "(", p_after, ")")) %>% 
  select(Variable=Disease, n_before, median_before, n_after, median_after)  

datatable(rownames = FALSE, extensions = 'Buttons',
  data %>% 
  count(Disease) %>% 
  mutate(p=percent(nn/sum(nn))) %>% 
  mutate(total_percent = paste0(nn," (", p, ")")) %>% 
  select(Disease, total_percent) %>% 
  full_join(DiseasesLocal  %>% select(Variable, median_local=median_after), by=c("Disease"="Variable")) %>% 
  left_join(BinregDiseaseDF, by=c("Disease"="Variable")) %>% 
  full_join(DiseasesState %>% select(Variable, median_state=median_after), by=c("Disease"="Variable"))
)
```

## 2.11 Compliance with IfSG amendment

```
datatable(rownames = FALSE, extensions = 'Buttons',data %>% 
  filter(gesetz_local=="NachGesetz") %>%
  filter(!is.na(korrekt_local)) %>% 
  count(korrekt_local) %>% 
  mutate(p=round(nn*100/sum(nn)))
)
```

## 2.12 Analysis of state reporting time

```
Univariable_Overall_state  <- data %>%
  filter(!is.na(delay_to_report_state)) %>% 
  group_by(gesetz_state) %>%
  summarise(
    median=quantile(delay_to_report_state, probs = c(0.5), na.rm=TRUE),
    q25 = quantile(delay_to_report_state, probs = c(0.25), na.rm=TRUE),
    q75 = quantile(delay_to_report_state, probs = c(0.75), na.rm=TRUE),
    n=n()) %>% 
  mutate(median = paste0(n, "_", median, " (", q25, " - ", q75,")")) %>% 
  select(gesetz_state, median) %>% 
  spread(key=gesetz_state, value=median) %>%
  separate(VorGesetz, into=c("n_before", "median_before"), sep="_") %>% 
  separate(NachGesetz, into=c("n_after", "median_after"), sep="_") %>%
  mutate(n_before=as.numeric(n_before)) %>% 
  mutate(p_before=round(n_before*100/sum(n_before))) %>%
  mutate(n_before = paste0(n_before, "(", p_before, ")")) %>% 
  mutate(n_after=as.numeric(n_after)) %>% 
  mutate(p_after=round(n_after*100/sum(n_after))) %>% 
  mutate(n_after = paste0(n_after, "(", p_after, ")")) %>% 
  mutate(Variable = "Overall") %>% 
  select(Variable, n_before, median_before, n_after, median_after)  

Univariable_BL_state  <- data %>%
  filter(!is.na(ReportingStateName)) %>% 
  filter(!is.na(delay_to_report_state)) %>% 
  group_by(gesetz_state, ReportingStateName) %>%
  summarise(
    median=quantile(delay_to_report_state, probs = c(0.5), na.rm=TRUE),
    q25 = quantile(delay_to_report_state, probs = c(0.25), na.rm=TRUE),
    q75 = quantile(delay_to_report_state, probs = c(0.75), na.rm=TRUE),
    n=n()) %>% 
  mutate(median = paste0(n, "_", median, " (", q25, " - ", q75,")")) %>% 
  select(gesetz_state, ReportingStateName, median) %>% 
  spread(key=gesetz_state, value=median) %>%
  separate(VorGesetz, into=c("n_before", "median_before"), sep="_") %>% 
  separate(NachGesetz, into=c("n_after", "median_after"), sep="_") %>%
  mutate(n_before=as.numeric(n_before)) %>% 
  mutate(p_before=round(n_before*100/sum(n_before))) %>%
  mutate(n_before = paste0(n_before, "(", p_before, ")")) %>% 
  mutate(n_after=as.numeric(n_after)) %>% 
  mutate(p_after=round(n_after*100/sum(n_after))) %>% 
  mutate(n_after = paste0(n_after, "(", p_after, ")")) %>% 
  select(Variable = ReportingStateName, n_before, median_before, n_after, median_after)  

Univariable_DayOfWeek_state  <- data %>%
  filter(!is.na(Weekday_state)) %>% 
  filter(!is.na(delay_to_report_state)) %>% 
  group_by(gesetz_state, Weekday_state) %>%
  summarise(
    median=quantile(delay_to_report_state, probs = c(0.5), na.rm=TRUE),
    q25 = quantile(delay_to_report_state, probs = c(0.25), na.rm=TRUE),
    q75 = quantile(delay_to_report_state, probs = c(0.75), na.rm=TRUE),
    n=n()) %>% 
  mutate(median = paste0(n, "_", median, " (", q25, " - ", q75,")")) %>% 
  select(gesetz_state, Weekday_state, median) %>% 
  spread(key=gesetz_state, value=median) %>%
  separate(VorGesetz, into=c("n_before", "median_before"), sep="_") %>% 
  separate(NachGesetz, into=c("n_after", "median_after"), sep="_") %>%
  mutate(n_before=as.numeric(n_before)) %>% 
  mutate(p_before=round(n_before*100/sum(n_before, na.rm=TRUE))) %>%
  mutate(n_before = paste0(n_before, "(", p_before, ")")) %>% 
  mutate(n_after=as.numeric(n_after)) %>% 
  mutate(p_after=round(n_after*100/sum(n_after))) %>% 
  mutate(n_after = paste0(n_after, "(", p_after, ")")) %>% 
  select(Variable = Weekday_state, n_before, median_before, n_after, median_after)  


EmptyLine<-data.frame(Variable=" ", n_before=" ", median_before=" ", n_after=" ", median_after=" ")


TableState <- rbind(Univariable_Overall_state, 
                       Univariable_BL_state , 
                       Univariable_DayOfWeek_state) 


datatable(rownames = FALSE, extensions = 'Buttons',TableState)
```

```
datatable(rownames = FALSE, extensions = 'Buttons',
  data %>% 
  filter(gesetz_state=="NachGesetz") %>%
  filter(!is.na(korrekt_state)) %>% 
  count(korrekt_state) %>% 
  mutate(p=round(nn*100/sum(nn)))
)
```

## 2.13 Analysis of notification to process time

```
datatable(rownames = FALSE, extensions = 'Buttons',
  data %>% 
  filter(!is.na(notificationProcessDelay)) %>% 
  group_by(gesetz_local) %>% 
  summarise(n=n(), 
            median=median(notificationProcessDelay),
            mn=round(mean(notificationProcessDelay),3),
            sd=round(sd(notificationProcessDelay),3)) %>% 
    mutate(se=round(sd/sqrt(n),2),
           LCI=round(mn+qnorm(0.025)*se,2),
           UCI=round(mn+qnorm(0.975)*se),2)
)
```

## 2.14 Quality and workload of data

### 2.14.1 Handling food or working in communal facility

```
datatable(rownames = FALSE, extensions = 'Buttons',
data %>% 
  group_by(gesetz_local) %>% 
  summarise(n_total=n(), n_notNA = sum(!is.na(Status_33_36_42)), p=n_notNA/n_total) %>% 
  mutate(LCI = p - 1.96*sqrt((p*(1-p))/n_total),
         UCI = p + 1.96*sqrt((p*(1-p))/n_total))
)
```

### 2.14.2 Cases with onset of disease

```
datatable(rownames = FALSE, extensions = 'Buttons',
data %>% 
  group_by(gesetz_local) %>% 
  summarise(n_total=n(), n_notNA = sum(!is.na(Erkrankungsbeginn)), p=n_notNA/n_total) %>% 
  mutate(LCI = p - 1.96*sqrt((p*(1-p))/n_total),
         UCI = p + 1.96*sqrt((p*(1-p))/n_total))
)
```

### 2.14.3 Mean number of versions

```
datatable(rownames = FALSE, extensions = 'Buttons',
  data %>% 
  group_by(gesetz_local) %>% 
  summarise(n=n(), MeanVersionen = mean(VersionNo, na.rm=TRUE), sd=sd(VersionNo, na.rm=T)) %>% 
  mutate(se=sd/sqrt(n),LCI=MeanVersionen+qnorm(0.025)*se,UCI=MeanVersionen+qnorm(0.975)*se) 
)
```

## 2.15 Figure 3

```
plotCommunity <- data %>% 
  filter(Yearmonth>=as.Date("2012-04-01")&Yearmonth<as.Date("2014-04-01")) %>% 
  group_by(Yearmonth) %>% 
  summarise(n_total=n(), n_notNA = sum(!is.na(Status_33_36_42)), p=n_notNA/n_total) %>% 
  mutate(LCI = p - 1.96*sqrt((p*(1-p))/n_total),
         UCI = p + 1.96*sqrt((p*(1-p))/n_total)) %>% 
  select(Yearmonth, p, LCI, UCI)  %>% 
  ggplot(aes(x=Yearmonth, y=p))  + 
  geom_line(stat="identity", color="darkgreen") + 
  geom_ribbon(aes(ymin=LCI, ymax=UCI), alpha = 0.3, fill="darkgreen") +
  theme_classic(base_size = 14) +
  theme(axis.text.x  = element_text(angle=45, hjust=1, vjust=1))+
  theme(legend.position="none") +
  scale_y_continuous(limits=c(0.1, 0.3)) +
  scale_x_date(date_break="2 months", date_labels="%Y - %m", name="time in months") +
  geom_vline(xintercept=as.numeric(as.Date("2013-03-01")), linetype="dashed")+
  labs(x="Time in months", y="cases with information on food \n  handling or community service (in %)")

plotOnset <- data %>% 
  filter(Yearmonth>=as.Date("2012-04-01")&Yearmonth<as.Date("2014-04-01")) %>% 
  group_by(Yearmonth) %>% 
  summarise(n_total=n(), n_notNA = sum(!is.na(Erkrankungsbeginn)), p=n_notNA/n_total) %>% 
  mutate(LCI = p - 1.96*sqrt((p*(1-p))/n_total),
         UCI = p + 1.96*sqrt((p*(1-p))/n_total)) %>% 
  select(Yearmonth, p, LCI, UCI) %>%
  ggplot(aes(x=Yearmonth, y=p))  + 
  geom_line(stat="identity", color="red") + 
  geom_ribbon(aes(ymin=LCI, ymax=UCI), alpha = 0.3, fill="red") +
  theme_classic(base_size = 14) +
  theme(axis.text.x  = element_text(angle=45, hjust=1, vjust=1))+
  theme(legend.position="none") +
  scale_y_continuous(limits=c(0.6, 0.9)) +
  scale_x_date(date_break="2 months", date_labels="%Y - %m", name="time in months") +
  geom_vline(xintercept=as.numeric(as.Date("2013-03-01")), linetype="dashed")+
  labs(x="Time in months", y="cases with onset \n  of disease (in %)")

PlotMeanAnzahlVersionen <- data %>% 
  filter(Yearmonth>=as.Date("2012-04-01")&Yearmonth<as.Date("2014-04-01")) %>% 
  group_by(Yearmonth) %>% 
  summarise(n=n(), MeanVersionen = mean(VersionNo, na.rm=TRUE), sd=sd(VersionNo, na.rm=T)) %>% 
  mutate(se=sd/sqrt(n),LCI=MeanVersionen+qnorm(0.025)*se,UCI=MeanVersionen+qnorm(0.975)*se) %>% 
  ggplot(aes(x=Yearmonth, y=MeanVersionen)) +
  geom_line(color="#FF9933") +
  geom_ribbon(aes(ymin=LCI, ymax=UCI), alpha = 0.3, fill="#FF9933") +
  theme_classic(base_size = 14) +
  theme(axis.text.x  = element_text(angle=45, hjust=1, vjust=1))+
  scale_y_continuous(limits=c(0.5,2.5), name="mean number of \n  versions of a case") +
  scale_x_date(date_break="2 months", date_labels="%Y - %m", name="time in months") +
  geom_vline(xintercept=as.numeric(as.Date("2013-03-01")), linetype="dashed")


figure3 <- grid.arrange(plotCommunity, plotOnset, PlotMeanAnzahlVersionen, ncol=1)
```

```
fileheight=8

setEPS()
postscript("figure3.eps", height = fileheight, width = fileheight*0.7, family = "ArialMT")
 grid.arrange(plotOnset, plotCommunity, PlotMeanAnzahlVersionen, ncol=1)
dev.off()
```

png 2

# 3 Part - Background information

## 3.1 Database query

```
if (!require("RODBC")) install.packages('RODBC'); require("RODBC")
# Creating a function for the query
getData <- function () {
  # hier wird definiert, wie man sich mit der Datenbank verbindet
  connection <- odbcDriverConnect("driver={SQL Server};server=sesql10;
                                  UID=SQL_SurvNet3_User;PWD=SurvNet3;
                                  Database=SurvNet3RKI2;",
                                  readOnlyOptimize=TRUE)

  query <- "
  SELECT
  CAST(DISEASE71.[OnsetOfDisease] AS DATE) AS [Erkrankungsbeginn]
  ,CAST(NA1.[DiagnosedAt] AS DATE) AS [ArztDiagnoseAm]
  ,CAST(NA1.[NotifiedAt] AS DATE) AS [ArztMeldungImGA]
  ,CAST(NL1.[DiagnosedAt] AS DATE) AS [LaborDiagnoseAm]
  ,CAST(NL1.[NotifiedAt] AS DATE) AS [LaborMeldungImGA]
  ,CAST(N3.[DiagnosedAt] AS DATE) AS [EigeneErmittlungDiagnoseAm]
  ,CAST(N3.[NotifiedAt] AS DATE) AS [EigeneErmittlungMeldungImGA]
  ,CAST(N4.[DiagnosedAt] AS DATE) AS [GemeinschaftP8DiagnoseAm]
  ,CAST(N4.[NotifiedAt] AS DATE) AS [GemeinschaftP8MeldungImGA]
  ,CAST(N5.[DiagnosedAt] AS DATE) AS [WeiterleitungAnderesGADiagnoseAm]
  ,CAST(N5.[NotifiedAt] AS DATE) AS [WeiterleitungAnderesGAMeldungImGA]
  ,CAST(N6.[DiagnosedAt] AS DATE) AS [GemeinschaftP34DiagnoseAm]
  ,CAST(N6.[NotifiedAt] AS DATE) AS [GemeinschaftP34MeldungImGA]
  ,CAST(N99.[DiagnosedAt] AS DATE) AS [AndereDiagnoseAm]
  ,CAST(N99.[NotifiedAt] AS DATE) AS [AndereMeldungImGA]
  ,CAST(Nx.[DiagnosedAt] AS DATE) AS [UnbekannteMeldeArtDiagnoseAm]
  ,CAST(Nx.[NotifiedAt] AS DATE) AS [UnbekannteMeldeArtImGA]
  ,CAST(RT1.[TrackedAt] AS DATE) AS [ErstelltGA]
  ,CAST(DISEASE71.[ReportingDate] AS DATE) AS [Meldedatum]
  ,CAST(RT2.[TrackedAt] AS DATE) AS [ExportGA]
  ,CAST(RT3.[TrackedAt] AS DATE) AS [ImportLS]
  ,CAST(RT4.[TrackedAt] AS DATE) AS [ExportLS]
  ,CAST(RT5.[TrackedAt] AS DATE) AS [ImportRKI]

  -- Disease --
  ,M.[GuiText] AS [Disease]
  ,MD.[SpecimenName]
  ,DisPat.[Pathogen]
  ,[Meta].[ParseCatalogeItem](10001, DISEASE71.[CaseDefCategoryComputed]) AS [CaseDefCategoryComputed]

  -- Software --
  ,SoftwareGA.[SN2SN3] AS 'SoftwareSN2SN3GA'
  ,SoftwareGA.[SW] AS 'SoftwareGruppeGA'

  -- Place --
  ,(SELECT I.ItemName FROM Meta.Catalogue2Item AS C2I INNER JOIN Meta.Item AS I ON C2I.IdItem = I.IdItem WHERE C2I.IdCatalogue = 1012 AND I.IdIndex = DISEASE71.[ReportingState])  AS [ReportingStateName]
  ,(SELECT ISNULL(TS.[Name1],'')+ ISNULL('-'+[Name2],'') FROM [Meta].[TransmittingSite] TS WHERE TS.[CodeSite] = V.[CodeRecordOwner]) AS [MeldendesGA]

  -- Person --
  ,(SELECT I.ItemName FROM Meta.Catalogue2Item AS C2I INNER JOIN Meta.Item AS I ON C2I.IdItem = I.IdItem WHERE C2I.IdCatalogue = 3002 AND I.IdIndex = DISEASE71.[Sex])  AS [Geschlecht]
  ,DISEASE71.[AgeComputed] AS [Alter]

  -- Variables --
  ,V.[VersionNo]
  ,Vacc.[StatusVaccination] AS 'ImpfungStatus'
  ,(SELECT I.ItemName FROM Meta.Catalogue2Item AS C2I INNER JOIN Meta.Item AS I ON C2I.IdItem = I.IdItem WHERE C2I.IdCatalogue = 1009 AND I.IdIndex = DISEASE71.[StatusPatientSetting]) AS 'Status_33_36_42'


  -- Time variables --
  ,RD.[Week] AS 'Meldewoche'
  ,RD.[Month] AS 'Meldemonat'
  ,RD.[Quarter] AS 'Meldequartal'
  ,RD.[WeekYear] AS 'Meldejahr'

  FROM
  [Data].[Version] AS V
  INNER JOIN [Data].[Disease71] AS DISEASE71 ON V.[IdVersion] = DISEASE71.[IdVersion]
  INNER JOIN Meta.DayTable RD ON RD.[IdDaySQL] = CAST(CAST(DISEASE71.[ReportingDate] AS FLOAT) AS INT)
  INNER JOIN Meta.[Type] M ON V.IdType = M.IdType
  INNER JOIN Meta.[Disease] MD ON V.IdType = MD.IdType AND V.IdSchema = MD.IdSchema
  OUTER APPLY [Data].[ExpandWithVaccination] (V.[IdVersion]) Vacc
  OUTER APPLY [Data].[ExpandWithDiseasePathogen](V.[IdVersion]) DisPat


  OUTER APPLY (SELECT [IdVersion], MIN([NotifiedAt]) AS [NotifiedAt], MIN([DiagnosedAt]) AS [DiagnosedAt] FROM [Data].[Disease71Notification] N WHERE V.[IdVersion] = N.[IdVersion] AND [NotificationType] = 1 GROUP BY [IdVersion]) NA1
  OUTER APPLY (SELECT [IdVersion], MIN([NotifiedAt]) AS [NotifiedAt], MIN([DiagnosedAt]) AS [DiagnosedAt] FROM [Data].[Disease71Notification] N WHERE V.[IdVersion] = N.[IdVersion] AND [NotificationType] = 2 GROUP BY [IdVersion]) NL1
  OUTER APPLY (SELECT [IdVersion], MIN([NotifiedAt]) AS [NotifiedAt], MIN([DiagnosedAt]) AS [DiagnosedAt] FROM [Data].[Disease71Notification] N WHERE V.[IdVersion] = N.[IdVersion] AND [NotificationType] = 3 GROUP BY [IdVersion]) N3
  OUTER APPLY (SELECT [IdVersion], MIN([NotifiedAt]) AS [NotifiedAt], MIN([DiagnosedAt]) AS [DiagnosedAt] FROM [Data].[Disease71Notification] N WHERE V.[IdVersion] = N.[IdVersion] AND [NotificationType] = 4 GROUP BY [IdVersion]) N4
  OUTER APPLY (SELECT [IdVersion], MIN([NotifiedAt]) AS [NotifiedAt], MIN([DiagnosedAt]) AS [DiagnosedAt] FROM [Data].[Disease71Notification] N WHERE V.[IdVersion] = N.[IdVersion] AND [NotificationType] = 5 GROUP BY [IdVersion]) N5
  OUTER APPLY (SELECT [IdVersion], MIN([NotifiedAt]) AS [NotifiedAt], MIN([DiagnosedAt]) AS [DiagnosedAt] FROM [Data].[Disease71Notification] N WHERE V.[IdVersion] = N.[IdVersion] AND [NotificationType] = 6 GROUP BY [IdVersion]) N6
  OUTER APPLY (SELECT [IdVersion], MIN([NotifiedAt]) AS [NotifiedAt], MIN([DiagnosedAt]) AS [DiagnosedAt] FROM [Data].[Disease71Notification] N WHERE V.[IdVersion] = N.[IdVersion] AND [NotificationType] = 999999 GROUP BY [IdVersion]) N99
  OUTER APPLY (SELECT [IdVersion], MIN([NotifiedAt]) AS [NotifiedAt], MIN([DiagnosedAt]) AS [DiagnosedAt] FROM [Data].[Disease71Notification] N WHERE V.[IdVersion] = N.[IdVersion] AND [NotificationType] IN (0,-1) GROUP BY [IdVersion]) Nx
  OUTER APPLY (SELECT [IdRecord], MIN([TrackedAt]) AS [TrackedAt] FROM [Data].[RecordTrack] RT WHERE V.[IdRecord] = RT.[IdRecord] AND [Action] = 1 GROUP BY [IdRecord]) RT1
  OUTER APPLY (SELECT [IdRecord], MIN([TrackedAt]) AS [TrackedAt] FROM [Data].[RecordTrack] RT WHERE V.[IdRecord] = RT.[IdRecord] AND [Action] = 2 AND [CodeSite] LIKE '1.__._%' GROUP BY [IdRecord]) RT2
  OUTER APPLY (SELECT [IdRecord], MIN([TrackedAt]) AS [TrackedAt] FROM [Data].[RecordTrack] RT WHERE V.[IdRecord] = RT.[IdRecord] AND [Action] = 3 AND [CodeSite] LIKE '1.__.' GROUP BY [IdRecord]) RT3
  OUTER APPLY (SELECT [IdRecord], MIN([TrackedAt]) AS [TrackedAt] FROM [Data].[RecordTrack] RT WHERE V.[IdRecord] = RT.[IdRecord] AND [Action] = 2 AND [CodeSite] LIKE '1.__.' GROUP BY [IdRecord]) RT4
  OUTER APPLY (SELECT [IdRecord], MIN([TrackedAt]) AS [TrackedAt] FROM [Data].[RecordTrack] RT WHERE V.[IdRecord] = RT.[IdRecord] AND [Action] = 3 AND [CodeSite] = '1.' GROUP BY [IdRecord]) RT5

  OUTER APPLY [Data].[ExpandWithSoftware] (V.[IdRecord]) SoftwareGA WHERE
  V.[IdRecordType] = 1    -- nur Faelle
  AND V.[IsCurrent] = 1   -- nur aktuelle Version des Falls
  AND V.[IsActive] = 1   -- nur aktive Faelle (nicht geloescht oder verworfen)
  AND DISEASE71.[ReportingDate] BETWEEN '2012-03-29' AND '2014-03-28' "
  data <- sqlQuery(connection, query)
  close(connection)
  data
}

# Executing the function
#data <- getData()

# Saving the dataset
#save(data, file="rawData.RData")
```

## 3.2 Session Info

```
sessionInfo()
```

R version 3.4.0 (2017-04-21) Platform: x86\_64-pc-linux-gnu (64-bit) Running under: Ubuntu 16.04.1 LTS

Matrix products: default BLAS: /usr/lib/libblas/libblas.so.3.6.0 LAPACK: /usr/lib/lapack/liblapack.so.3.6.0

locale: [1] LC\_CTYPE=de\_DE.UTF-8 LC\_NUMERIC=C  
[3] LC\_TIME=de\_DE.UTF-8 LC\_COLLATE=de\_DE.UTF-8  
[5] LC\_MONETARY=de\_DE.UTF-8 LC\_MESSAGES=de\_DE.UTF-8  
[7] LC\_PAPER=de\_DE.UTF-8 LC\_NAME=C  
[9] LC\_ADDRESS=C LC\_TELEPHONE=C  
[11] LC\_MEASUREMENT=de\_DE.UTF-8 LC\_IDENTIFICATION=C

attached base packages: [1] grid stats graphics grDevices utils datasets methods  
[8] base

other attached packages: [1] MASS\_7.3-45 DT\_0.2 svglite\_1.2.0  
[4] timeDate\_3012.100 gridExtra\_2.2.1 knitr\_1.16  
[7] scales\_0.4.1 ggplot2\_2.2.1 tidyr\_0.6.3  
[10] dplyr\_0.5.0

loaded via a namespace (and not attached): [1] Rcpp\_0.12.11 magrittr\_1.5 munsell\_0.4.3 colorspace\_1.3-2 [5] R6\_2.2.1 rlang\_0.1.1 stringr\_1.2.0 plyr\_1.8.4  
[9] tools\_3.4.0 gtable\_0.2.0 DBI\_0.6-1 htmltools\_0.3.6 [13] yaml\_2.1.14 lazyeval\_0.2.0 assertthat\_0.2.0 rprojroot\_1.2  
[17] digest\_0.6.12 tibble\_1.3.3 htmlwidgets\_0.8 evaluate\_0.10  
[21] rmarkdown\_1.5 labeling\_0.3 stringi\_1.1.5 compiler\_3.4.0  
[25] gdtools\_0.1.4 backports\_1.1.0 jsonlite\_1.5
